# HIV interventions across the care continuum for adolescents in high-burden countries: a systematic review and meta-analysis

**DOI:** 10.1016/j.eclinm.2024.102989

**Published:** 2024-11-28

**Authors:** Yusha Tao, Margaret Byrne, Dorian Ho, Zixuan Zhu, Jamie L. Conklin, Takhona G. Hlatshwako, Liyuan Zhang, Ogechukwu Benedicta Aribodor, Malida Magista, Wenjie Shan, Ujunwa F. Onyeama, Onyekachukwu Anikamadu, Linet M. Mutisya, Kelechi Chima, Ashiru Mustapha, Kathleen Agudelo Paipilla, Ijeoma Omenugha, Eleanor Namusoke Magongo, Juliet lwelunmor, Susan Vorkorper, Rachel Sturke, Weiming Tang, Damilola Walker, Joseph D. Tucker

**Affiliations:** a University of North Carolina Project-China, Guangzhou, China; b Dermatology Hospital of South Medical University, Guangzhou, China; c Institute of Global Health and Infectious Diseases, University of North Carolina at Chapel Hill, Chapel Hill, USA; d Department of Health Policy and Management, Gillings School of Global Public Health, University of North Carolina at Chapel Hill, Chapel Hill, USA; e Health Sciences Library, University of North Carolina at Chapel Hill, Chapel Hill, NC, USA; f Department of Social Policy and Intervention, University of Oxford, UK; g Social Innovation in Health Initiative (SIHI), Nnamdi Azikiwe University, Awka, Nigeria; h Department of Zoology, Nnamdi Azikiwe University, Awka, Nigeria; i Center for Tropical Medicine, Faculty of Medicine, Public Health and Nursing, Universitas Gadjah Mada, Indonesia; j Department of International Clinic, Shanghai Children's Medical Center, Shanghai Jiao Tong University School of Medicine, Shanghai, China; k Department of Health Behavior, University of North Carolina at Chapel Hill, Chapel Hill, NC, USA; l Brown School of Social Work, Washington University in St. Louis, St Louis, MO, United States; m Maternal and Child Health Project, Swedish Organization for Global Health, Mayuge, Uganda; n Nuffield Department of Medicine, University of Oxford, UK; o Faculty of Pharmacy, University of Ibadan, Ibadan, Nigeria; p Centro Internacional de Entrenamiento e Investigaciones Médicas, Cali, Colombia; q Barts and The London School of Medicine and Dentistry Queen Mary University of London, UK; r Ministry of Health, Kampala, Uganda; s School of Medicine/Division of Infectious Diseases, Washington University School of Medicine, St. Louis, MO, USA; t Fogarty International Center, National Institutes of Health, Bethesda, MD, USA; u HIV/AIDS Section, Health Programmes, United Nations Children's Fund, New York, USA; v Clinical Research Department, Faculty of Infectious and Tropical Diseases, London School of Hygiene and Tropical Medicine, London, UK

**Keywords:** Adolescent, HIV, Prevention-to-care cascade, Social and mental health outcomes, Adolescent engagement

## Abstract

**Background:**

Adolescents (10–19 years old) have poor outcomes across the prevention-to-treatment HIV care continuum, leading to significant mortality and morbidity. We conducted a systematic review and meta-analysis of interventions that documented HIV outcomes among adolescents in HIV high-burden countries.

**Methods:**

We searched PubMed, EMBASE, Scopus, and the Cochrane Library for studies published between January 2015 and September 2024, assessing at least one HIV outcome along the prevention-to-care cascade, including PrEP uptake, HIV testing, awareness of HIV infections, ARV adherence, retention, and virological suppression. We included studies from 37 HIV high-burden countries evaluating interventions with a comparator group. Random-effects meta-analysis was used to pool the effects of the interventions on study outcomes. While the primary focus was on outcomes related to the HIV care cascade, we also evaluated social outcomes and mental health outcomes when sufficient data were available. Adolescent engagement in studies was assessed using Hart's ladder. The study was registered in PROSPERO, CRD42024569203.

**Findings:**

We identified 12,411 unique records, of which 99 were included in the final analysis, comprising 57 randomized controlled trials and 42 non-randomized studies, with a total sample size of 441,252 participants. Our analysis found that asset-building interventions significantly improved HIV testing uptake (three studies, RR: 1.38, 95% CI 1.15–1.65) compared to control conditions. Differentiated service delivery interventions were associated with improved ART retention (five studies, RR: 1.18, 95% CI 1.04–1.36) and virological suppression (seven studies, RR: 1.19, 95% CI 1.06–1.33) compared to control conditions. Financial incentives significantly enhanced ART adherence (two studies, RR: 1.52, 95% CI 1.23–1.89) compared to control conditions. Digital interventions, such as mobile phone applications and telehealth services, significantly increased HIV testing uptake (two studies, RR: 1.79, 95% CI 1.23–2.61) compared to control conditions. Positive impacts were also observed for these interventions on social outcomes. Most studies adopted minimal to moderate adolescent engagement. For HIV testing, a stronger effect was seen in studies with moderate to substantial adolescent engagement, yielding an RR of 2.37 (95% CI: 1.43–3.93; nine studies), compared to a lower RR of 1.23 (95% CI: 1.15–1.31; 13 studies) in studies with minimal engagement. Notably, the strength of evidence is moderate to low due to the considerable heterogeneity across studies and limited included studies.

**Interpretation:**

Our data demonstrate several evidence-based interventions that can enhance adolescent HIV outcomes across the care continuum. Our findings are relevant in many HIV high-burden countries and can help to inform national and regional adolescent HIV policy.

**Funding:**

This study was supported by 10.13039/100006641UNICEF, the Adolescent HIV Prevention and Treatment Implementation Science Alliance (AHISA), and the US 10.13039/100000002NIH (NIAID K24AI143471, NICHD 1UG1HD113156, UM2HD116395).

**Research in context**

**Evidence before this study**

We performed a systematic search for articles on interventions to improve HIV and related outcomes among adolescents aged 10–19 in high-burden HIV countries, published between January 1, 2015, and September 27, 2024. The existing literature highlights the disparities in HIV outcomes among adolescents, with lower rates of diagnosis, treatment, and viral suppression compared to older populations. However, there has been a lack of reviews on interventions for adolescents in high-burden settings that demonstrate effects across multiple domains of health and well-being.

**Added value of this study**

Our study synthesizes evidence from 99 studies assessing interventions improving adolescent outcomes along the extended HIV cascade. We highlight several promising intervention types, such as differentiated service delivery, multi-level interventions, financial incentives, digital interventions, and asset building, that address both HIV-specific and broader social and mental health outcomes. Our findings provide actionable insights for policymakers on effective resource allocation in the context of constrained and declining financing for health.

**Implications of all the available evidence**

Adolescents continue to face disproportionate challenges related to HIV. The evidence in this review can be used to inform scaling up interventions to achieve 95-95-95 targets and address social determinants of health. Policymakers and program implementers should prioritize investing in further evidence generation as promising interventions are scaled up, ensuring that further research addresses existing gaps to better support adolescents in high-burden HIV countries and improve their HIV-related outcomes.

## Introduction

The Universal Test and Treat (UTT) strategy introduced by the World Health Organization (WHO) in 2015 has helped fast-track national HIV responses towards epidemic control. Notably, more than 60 countries are close to achieving either the 90-90-90 targets or their successor, the 95-95-95 targets, which were adopted by Member States resolution in 2015 and 2021, respectively.^1^ As of 2023, the global estimates indicate that 86% of all people with HIV know their status, 89% of people who know their status are on treatment, and 93% of those on treatment are virally suppressed. However, there are weak spots in achievement—both HIV incidence and mortality have not reduced at rates fast enough to achieve any of the global targets, and entire sub-populations experience inequitable outcomes, notably key populations, their partners, and adolescents and young people.

Globally, there are an estimated 2.4 million adolescents (10–19 years old) with HIV, and 250,000 new infections occur in this age group annually. In Africa, where over 90% of adolescents with HIV reside, HIV remains the leading cause of mortality among adolescents. Adolescent girls 15–19 in Africa bear a staggering 90% of new HIV infections in this age group. These adolescents are recognized to have suboptimal HIV outcomes across the entire prevention-to-treatment continuum, starting with primary prevention and (early) diagnosis, onwards to linkage-to-care, ART adherence, and viral suppression.^2^ There is lower attainment across the cascade for younger cohorts than older populations. Specifically, the estimated attainment stands at 60-49-81 for individuals aged 15–24 years, in contrast to 70-63-91 for those older than 25.^1^^,^^3^ As a result, high-burden HIV countries will require investment in aggressive scale-up of strategies to simultaneously improve the clinical outcomes of adolescents while reducing new HIV infections.

Given their precarious positioning neither fully in childhood nor entirely in adulthood, our knowledge of how to deliver for adolescents at scale is still in its infancy. Adolescents have limited autonomy in making their own health decisions, and low adolescent engagement with decision-makers exacerbates their underrepresentation in research and widening knowledge gaps.^4^ Many studies exclude adolescents because of the legal and political context,^5^ further perpetuating this gap.^6^, ^7^, ^8^ Consequently, very few jurisdictions have systems that operate at scale to meet adolescents' integrated health and social needs. However, there is a growing set of options for adolescents with and affected by HIV,^9^ with some documentation from high-income countries and upper-middle-income countries where there is a lower burden of HIV infection.^10^^,^^11^

What is clear is that despite the known shortcomings, there is a never-before opportunity to advance adolescent health and well-being outcomes: investment in the leadership, agency, and well-being of young people is now a primary agenda at the highest levels of global cooperation.^12^ Buoyed by the unprecedented opportunity to reimagine the global experience of the world's largest cohort of young people—estimated at 1.8 billion—key mandate-holders for young people, including UNICEF, WHO, and Member States, have proposed a framework that advances adolescent well-being holistically, with Member States endorsement.^13^ This political commitment arrives as decades of investment in product research and development avail a greater array of options for both treatment and prevention of HIV—alongside multi-objective technologies that address other conditions (notably contraception). Finally, there has been recognition in many quarters that we need dedicated efforts to fast-track the inclusion of this population into the appropriate phases of HIV research, from the basic and clinical research (pre-market) phases to implementation research post-marketing.^14^, ^15^, ^16^

The primary objective of this systematic review and meta-analysis is to examine the literature to identify the program models and interventions that have been effective at improving HIV and related outcomes among adolescents in high-burden HIV countries and to synthesize the evidence from these. We also assess the extent of adolescent engagement in HIV intervention research conducted across these high-burden HIV countries. This review assesses the state of our knowledge and evidence for adolescents across the prevention-to-treatment cascade. Our review also complements a parallel effort to engage young innovators, makers, and activists in a crowdsourcing effort to understand youth perspectives on the types of innovation, solutions, and investments that might enhance their experiences in care and, thus, their outcomes at scale. This review's evidence helps assess the potential for effectiveness and impact of a curated list of youth-defined solutions being considered for their merit as part of the global policymaking processes.

## Methods

This study is registered in PROSPERO CRD42024569203. This systematic review and meta-analysis applied the guidelines in the *Cochrane Handbook of Systematic Reviews of Interventions* and is reported following *Preferred Reporting Items for Systematic Reviews and Meta-Analyses*,^17^ *the Meta-analyses Of Observational Studies in Epidemiology checklist*,^18^ and *Guidelines for Accurate and Transparent Health Estimates Reporting*.^19^ We employed a comprehensive search strategy across databases, supplemented by manual searches and expert consultation. Study selection included initial automation-assisted and subsequent manual screening, with machine learning aiding. This was followed by data extraction, adolescent engagement categorization, and risk of bias assessment. A random-effects meta-analysis was applied to pool the effects of the interventions on study outcomes.

### Ethics

Given that this study is a systematic review and meta-analysis that synthesizes and analyzes previously published content, ethical approval was not required.

### Inclusion criteria

#### Study design

We initially included studies that were published between 2015/01/01 and 2023/06/01. The search was updated on 2024/09/27. We chose 2015 as a watershed year because of the introduction of landmark global normative recommendations (UTT, Option B+, Differentiated Service Delivery), coupled with concerted efforts to accelerate the inclusion of this age group in research as a top priority.^20^ We included studies with a comparator arm, including randomized controlled trials (RCTs) and controlled non-randomized studies. Non-randomized studies included historically controlled studies, interrupted time series studies, cohort studies, controlled before-and-after studies, non-randomized controlled studies, and non-randomized cluster-controlled studies.

#### Populations

We adopted the WHO and UNAIDS standard definition of adolescents being individuals 10–19 years of age; WHO clinical guidelines for pediatric HIV define children as up to early adolescents (10–14). In cases where adolescents were a nested subset of a larger study, the study or sub-analysis had to report on the effectiveness of HIV interventions in a population with more than 75% of adolescents (age 10–19) with or affected by HIV. Studies may have reported on any or all of the following groupings: 10–14, 15–19, 11–18, 10–19.

#### Geographic scope

We identified 37 high-burden countries contributing to more than 75% of the global burden of adolescents with HIV based on their contribution to the total number of estimated adolescent HIV incident cases and the total number of adolescents with HIV. These countries are: Angola, Bangladesh, Botswana, Brazil, Burundi, Cameroon, Chad, China, Côte d'Ivoire, the Democratic Republic of the Congo, Djibouti, Dominican Republic, Eswatini, Ethiopia, Ghana, Haiti, India, Indonesia, Islamic Republic of Iran, Kenya, Lesotho, Malawi, Mozambique, Myanmar, Namibia, Nigeria, Pakistan, Papua New Guinea, Philippines, Rwanda, South Africa, Uganda, Ukraine, United Republic of Tanzania, Uzbekistan, Zambia, and Zimbabwe. Studies had to be conducted exclusively in these countries. If a study was conducted in both a high-burden country and another country (e.g., the US and South Africa), it was included only if the results for the high-burden country could be separately analyzed and reported.

#### Interventions

This systematic review includes studies assessing the effectiveness of programs and/or service delivery models that aim to influence adolescent behaviors and/or clinical outcomes related to HIV. Studies may be conducted in any setting, delivered through an array of channels, and deployed through a diverse health workforce for delivery. Interventions may address multiple points across the prevention-to-treatment care cascade and operate at multiple socio-ecological levels (i.e., targeting the individual, peers, social networks, and systems). We also aimed to codify adolescent engagement in the intervention design and delivery using a published youth engagement typology/scale known as Hart's ladder (detailed below).^21^^,^^22^

#### Comparator

Interventions or service-delivery models as described above may be compared to a standard of care, an alternative package of care/intervention, or no package of care/intervention.

#### Outcomes

Given our interest in more recent evidence on adolescent HIV outcomes and awareness of other comprehensive reviews focused on discrete aspects of HIV prevention, we limited outcomes of interest to the adolescent treatment cascade and ARV-based prevention (oral pre-exposure prophylaxis) specifically. Since structural factors, such as social, economic, and legal conditions, play a crucial role in HIV prevention and treatment,^23^ and an emerging body of scientific evidence suggests that addressing these factors is critical for a comprehensive HIV response from prevention through to treatment,^24^ we also included these outcomes in our analysis.

### Search strategy

#### Search terms

The search strategy was developed in consultation with a health sciences librarian (JC) at the University of North Carolina at Chapel Hill. The search included subject headings and keywords for three main concepts: “adolescents”, “HIV”, and geography terms for the 37 countries (Appendix pp 3–7). In addition, the Cochrane highly sensitive filter for PubMed randomized trials was applied to the search.^25^ The filter was expanded to include other clinical trial designs and adapted for the other databases. There were no language restrictions. Publications not in English were translated to English using Google Translate to assess for study eligibility. Studies were excluded if they were mainly qualitative, reported social determinants and other factors only, and/or described implementation processes only.

#### Sources

A systematic search was conducted on June 1, 2023, and updated on September 27, 2024, to identify eligible studies in the following databases: PubMed, EMBASE, Scopus, and the Cochrane Library. Reference lists of included papers were manually searched for additional relevant citations. The ‘cited by’ tool in Google Scholar was also used to identify potentially relevant studies. We also screened and searched the references of the related recent systematic reviews. Further, if we required more information to confirm the eligibility of a study, we contacted the authors by email.

#### Data management and study selection

We initially identified 10,711 references from electronic databases, which were imported into EndNote, and duplicates were removed. To enhance screening efficiency, we used automation techniques through ICF's Document Classification and Topic Extraction Resource (DoCTER) (https://www.icf-docter.com/) to prioritize references by relevance. Previous literature has indicated that the use of this supervised clustering with the ensemble learning approach results in a 95% recall rate of relevant studies,^26^ of which we found suitable for our systematic review.

The screening process occurred in two stages. First, two researchers (YT and LZ) independently screened a batch of 294 references from the total pool. Of those, 48 studies were moved to full-text screening and were used as seed studies to identify similarly relevant articles through supervised clustering. Using DoCTER, references were assigned an ensemble score of 0 through 6, reflecting their relevance across six models (thematic relevance, methodology, population characteristics, intervention types, geographical focus, and methodological rigor) used during the supervised clustering stage. Subsequently, 1199 studies with a score of five or six were imported into Covidence systematic review software (Veritas Health Innovation, Melbourne, Australia, available at www.covidence.org) for further screening. Two researchers (either YT, LZ, ZZ, DH, OA, WS, UO, OA, MM, KA, JT, or IO) independently screened each title and abstract using a customized checklist for study selection. Conflicts were resolved through discussion or arbitration with a third reviewer (YT, JT, LZ, ZZ, DH). In the second screening stage, studies deemed both relevant and irrelevant through the first stage were used as training data for machine learning. Again, using DoCTER, each study not yet screened was assigned a probability score that ranged from 0 to 1, from less to more likely relevant. To address potential false negatives, we closely monitored the results from the machine learning model. Similarly, two researchers (YT, DH) independently screened each title and abstract until relevance dropped off at a probability score of 0.45, ensuring that potentially relevant studies were not overlooked. Finally, 762 studies were included for full-text screening.

Two reviewers (YT, DH) independently assessed full-text versions of selected abstracts to ensure that inclusion criteria were met. Like before, conflicts were resolved through discussion or arbitration with a third researcher (BM). Specific reasons for study exclusion were documented and reported using the Preferred Reporting Items for Systematic Reviews and Meta-Analyses (PRISMA) flow diagram.

### Extraction

Data from the full text of selected studies was extracted by one reviewer and checked by a second reviewer (either YT, DH, LZ, ZZ, BM, IA, OBA, OA, MM, or UO). The extraction form was pre-tested with five studies. We extracted data on participant characteristics, study and intervention design, and our primary outcomes: the impact of interventions on the uptake of PrEP, HIV testing, awareness of HIV infections, ARV adherence and retention, and virological suppression. We also extracted social outcomes, such as school dropout, experiences of violence, and stigma reduction, and mental health outcomes, including SSQ (Shona Symptoms Questionnaire), SDQ (Strengths and Difficulties Questionnaire), and PHQ (Patient Health Questionnaire), where available. Interventions assessed in each study were extracted to identify all intervention types. Intervention types were categorized into an inductively generated list of discrete intervention descriptors (Appendix pp 8 and 9). Specifically, digital interventions included using online platforms, telehealth, telemedicine, wearables, connectivity, mobile applications, personalized care, and other internet-enabled tools to provide adolescents with health information, support services, feedback elicitation, and engagement. Mobile health interventions (m-health) are a subset of these, referring to the use of mobile devices such as smartphones and tablets to deliver health-related messages, reminders, and support for treatment adherence through SMS, apps, and other mobile technologies. Asset-building interventions focused on enhancing the social, financial, and educational resources available to adolescents, such as skills training programs, financial literacy education, initiatives to improve their access to education and employment opportunities, and related. Additionally, financial incentives typically involve direct monetary rewards aimed at achieving specific health outcomes, such as adherence to antiretroviral therapy, while economic strengthening encompasses broader strategies designed to improve an individual's overall economic stability, which may indirectly support adherence through enhanced financial security. When the intervention in the control or comparator group differed from the standard of care for that setting, the information on the comparator intervention was extracted. When multiple interventions were implemented simultaneously or delivered at multiple ecological levels (e.g., individual, family, community-wide, healthcare system), the study was categorized as a multi-level intervention. All intervention extractions were conducted in duplicate by reviewers YT and DH, with any inconsistencies resolved by a third reviewer (BM).

### Categorizing adolescent engagement

We used Hart's ladder to categorize levels of adolescent engagement following Hart's typology, which describes different degrees of youth involvement in projects or programs.^22^ We adapted Hart's ladder to classify adolescent engagement based on the level of decision-making of adolescents in the research study: substantial, moderate, minimal, or no engagement.^4^^,^^21^ Substantial engagement was defined as research activities initiated and led by adolescents, with adults facilitating an enabling environment or contributing relevantly, while adolescents retain significant decision-making power and leadership opportunities. Moderate engagement involved activities initiated by adults with shared decision-making between adolescents and adults. Minimal engagement was defined as adolescents being consulted for opinions, assigned specific roles, or kept informed about research events without decision-making power. No engagement was defined as the absence of participatory approaches or activities in the research.

### Assessment of risk of bias and quality of evidence

We used version 2 of the Cochrane Risk of Bias tool to assess the risk of bias for RCTs.^27^^,^^28^ The tool considers the following domains of bias: bias arising from the randomization process, bias due to deviations from intended interventions, bias due to missing outcome data, bias in the measurement of the outcome, and bias in the selection of the reported result. All selected studies were scored as either low, some concerns, or high risk of bias (Appendix pp 10 and 11). To assess the risk of bias in non-randomized studies of interventions, we used the ROBINS-I and the tools developed by Hoy and colleagues.^29^ The tool considers four domains of bias: selection bias, bias due to missing outcome data, bias in the measurement of the outcome, and bias in the analysis. Studies were scored across domains and reported as having low, some/medium, or high risk of bias (Appendix p 12). Two reviewers (either BM, ZZ, DH, MM, or OA) did a risk of bias assessment using those tools, and any discrepancies were discussed with a third reviewer (either BM or DH). The quality of evidence was assessed using the Grading of Recommendations Assessment, Development, and Evaluation framework, considering the risk of bias, consistency of results, the evidence's directness, the estimates' precision, and reporting bias.^30^ Based on the evaluations of these domains, we graded the certainty for each body of evidence as high, moderate, low, or very low.

### Statistics

We used random-effects meta-analysis to synthesize the pooled outcome measure estimates. Given the predominance of RCTs in our data, we primarily calculated risk ratios (RRs) and 95% CIs to assess the association between each intervention and the outcomes across the HIV care cascade. Where necessary, we converted ORs to RRs to ensure consistency in evaluating cascade outcomes. For continuous outcomes related to stigma and psychological variables, we used standardized mean differences (SMDs) to capture effect sizes. We used a regression-based Egger test to assess publication bias for each outcome. Heterogeneity across studies was measured with the I^2^ statistic, with an I^2^ of less than 25% representing low heterogeneity, an I^2^ of 25–75% representing moderate heterogeneity, and an I^2^ of more than 75% representing high heterogeneity. Two-sided p-values of less than 0.05 were deemed to be statistically significant. All analyses were done with Stata, version 15.0.

We used thematic analyses to summarize textual data describing various adolescent engagement activities employed in identified research studies, which were then classified using a conceptual framework based on modified Hart's ladder. Two researchers (either BM, DH, or YT) independently analyzed textual data into four categories. Engagement activities identified were then categorized and independently coded once for each intervention as per the modified Hart's ladder described above. Each study was given a score for the degree of engagement based on the coded engagement activities.

### Role of funding source

The funder of the study had no role in study design, data collection, data analysis, or data interpretation for this report.

## Results

**Fig. 1:**
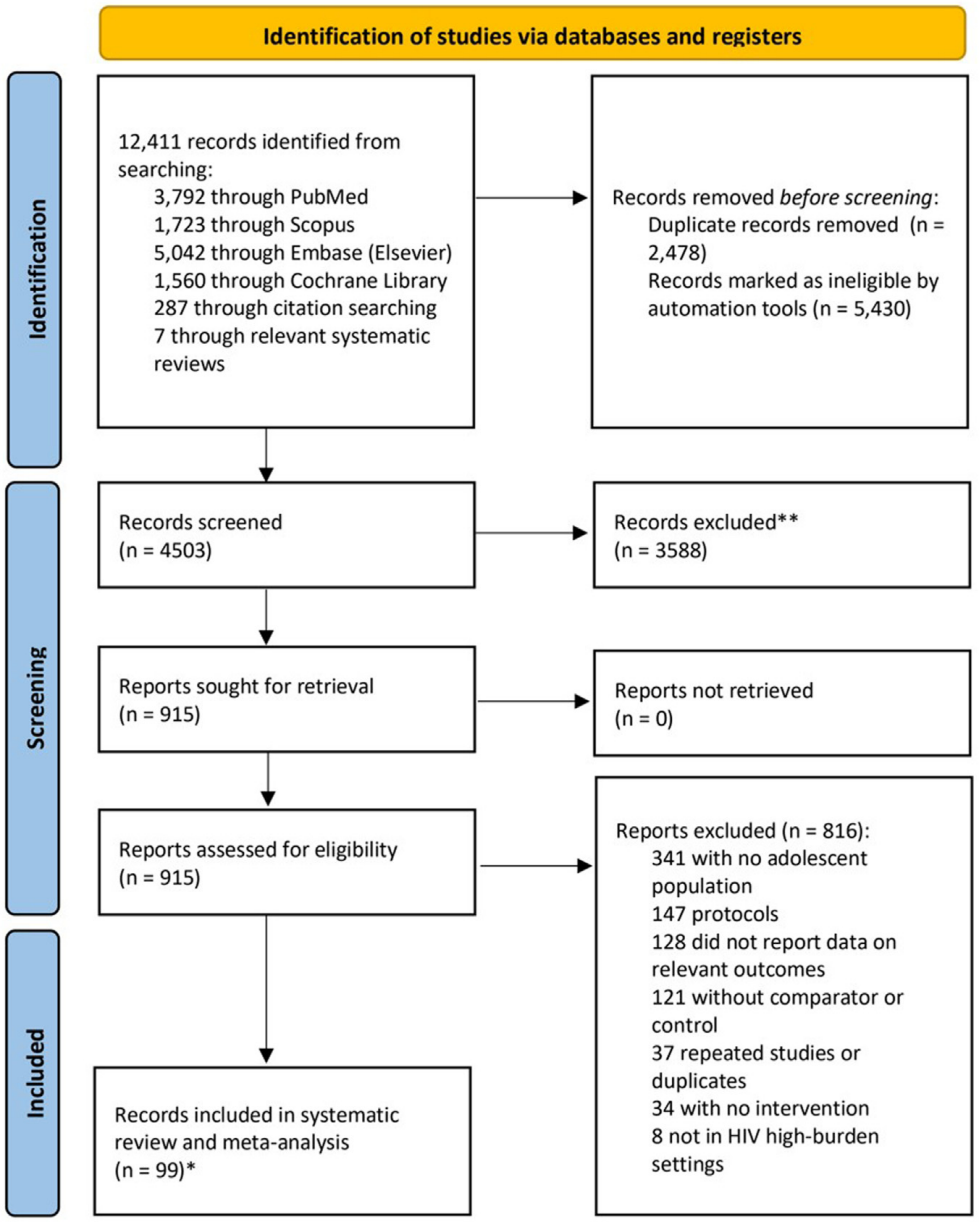
Flow diagram of study inclusion. A total of 99 studies were included in the overall analysis. (A) The first search phase covered studies published between January 1, 2015, and July 31, 2023; (B) The second search phase extended from August 1, 2023, to September 27, 2024. ∗Initially, 77 studies were included from the original search. In this updated round, two abstracts (by Nair G and Ngure K) were replaced, as Nair G's full manuscript was published in 2023 and included in the second search.

10,711 citations from databases and seven from other sources were identified during the initial search period from January 1, 2015, to June 1, 2023, and a further 1692 were identified when the search was extended to September 28, 2024. When duplicates were removed, 4465 titles (3204 from the initial search and 1261 from the extended search) remained for screening. After the abstract and full-text screening, 99 unique records, comprising 96 journal articles and three conference abstracts, were included in our systematic review and meta-analysis (Fig. 1, Table 1).^31^, ^32^, ^33^, ^34^, ^35^, ^36^, ^37^, ^38^, ^39^, ^40^, ^41^, ^42^, ^43^, ^44^, ^45^, ^46^, ^47^, ^48^, ^49^, ^50^, ^51^, ^52^, ^53^, ^54^, ^55^, ^56^, ^57^, ^58^, ^59^, ^60^, ^61^, ^62^, ^63^, ^64^, ^65^, ^66^, ^67^, ^68^, ^69^, ^70^, ^71^, ^72^, ^73^, ^74^, ^75^, ^76^, ^77^, ^78^, ^79^, ^80^, ^81^, ^82^, ^83^, ^84^, ^85^, ^86^, ^87^, ^88^, ^89^, ^90^, ^91^, ^92^, ^93^, ^94^, ^95^, ^96^, ^97^, ^98^, ^99^, ^100^, ^101^, ^102^, ^103^, ^104^, ^105^, ^106^, ^107^, ^108^, ^109^, ^110^, ^111^, ^112^, ^113^, ^114^, ^115^, ^116^, ^117^, ^118^, ^119^, ^120^, ^121^, ^122^, ^123^, ^124^, ^125^, ^126^, ^127^, ^128^, ^129^ These records included 57 RCTs and 42 non-randomized studies. The most common study designs were cluster randomized trials (n = 30), RCTs (n = 27), cohort studies (n = 14), non-randomized controlled trials (n = 11), and controlled before-and-after studies (n = 11). Twenty-third studies (23.2%) were in low-income countries, 51 (51.5%) studies were in lower-middle-income countries, and eighteen studies (18.2%) were in upper-middle-income countries. Our study populations included adolescents with HIV (46 [46.5%]), general adolescents living in high HIV-burden contexts (22 [22.2%]), vulnerable and orphaned adolescents (19 [19.2%]), adolescents at-risk of HIV (5 [5.1%]), perinatally adolescents living with HIV (4 [4.0%]), pregnant and parenting adolescents (2 [2.0%]), and transgender adolescent women (1 [1.0%]). The primary types of interventions identified through this search included differentiated service delivery (15 [15.2%]), psychosocial support (12 [12.1%]), multi-level interventions (9 [9.1%]), economic strengthening (7 [7.1%]), educational interventions (7 [7.1%]), m-health (7 [7.1%]), mental health interventions (6 [6.1%]), asset building (6 [6.1%]), treatment as prevention (5 [5.1%]), financial incentives (5 [5.1%]), digital interventions (5 [5.1%]), self-care interventions (3 [3.0%]), school support (3 [3.0%]), disclosure support (2 [2.0%]), clinical quality improvement (2 [2.0%]), targeted contact-based HIV testing interventions (2 [2.0%]), novel prevention options (1 [1.0%]), point-of-care viral load testing and targeted drug resistance mutation testing (1 [1.0%]) and prevention of mother-to-child transmission (1 [1.0%]) (Table 2). Three studies (3.0%) focused on HIV prevention, 91 studies (91.9) focused on HIV treatment, and five (5.1%) included both prevention and treatment. HIV outcomes included PrEP uptake, HIV testing, awareness of HIV infections, ARV adherence, retention, and virological suppression.

**Table 1:** Characteristics of included studies (n = 99).

Study	Study design	Population	Age group	Gender (% Females)	Country	Setting	Intervention category	Prevention-to-treatment care cascade outcomes
Abiodun O, 2021^31^	Randomized controlled trial	Adolescents with HIV	Older adolescents	48.3	Nigeria	HIV/STD clinics	M-Health	ART adherence, viral suppression, pill count, and ACTG scores
Abili M, 2023^32^	Cohort study	Adolescents with HIV	Older adolescents	70	Malawi	HIV/STD clinics	Differentiated service delivery	Viral suppression
Amstutz A, 2020^33^	Cluster randomized controlled trial	General adolescents	Older adolescents	53.3	Lesotho	Community and population-based	Self-care interventions	HIV testing
Amzel A, 2018^34^	Non-randomized controlled trial	Adolescents with HIV	N/A	N/A	Lesotho	Community and population-based	Differentiated service delivery	Twelve-month ART retention, viral load coverage, and viral suppression
Aninanya GA, 2015^35^	Cluster randomized controlled trial	General adolescents	Older adolescents	41.1	Ghana	Community and population-based	Multi-level intervention	HIV counseling and testing
Arije O, 2023^36^	Non-randomized controlled trial	Vulnerable and orphaned adolescents	N/A	100	Nigeria	Community and population-based	Multi-level intervention	HIV testing
Barker D, 2019^37^	Non-randomized controlled trial	Perinatally adolescents living with HIV	Early adolescents	50	Ghana	HIV/STD clinics	Psychosocial support	Clinic attendance, WHO clinical staging, complete blood count, CD4 count, viral load, and ART resistance
Barnabee G, 2023^38^	Cohort study	Vulnerable and orphaned adolescents	Older adolescents	100	Namibia	Community and population-based	Differentiated service delivery	Persisted with PrEP at Month-1 visit
Beck-Sague CM, 2015^39^	Non-randomized controlled trial	Perinatally adolescents living with HIV	Early adolescents	66.3	Dominican Republic, Haiti	HIV/STD clinics	Disclosure support	Viral suppression, median viral load
Bermudez LG, 2018^40^	Cluster randomized controlled trial	Adolescents with HIV	Early adolescents	56	Uganda	HIV/STD clinics	Economic strengthening	Viral suppression, viral load
Birdthistle I, 2022^41^	Randomized controlled trial	Vulnerable and orphaned adolescents	Older adolescents	100	Kenya, South Africa	Community and population-based	Asset building	Awareness of HIV infections, HIV testing
Birdthistle I, 2022^42^	Non-randomized controlled trial	General adolescents	Older adolescents	60.8	South Africa	Online	Digital interventions	Awareness of HIV infections, HIV self-testing
Brathwaite R, 2022^43^	Cluster randomized controlled trial	Adolescents with HIV	Early adolescents	56	Uganda	General health facilities	Economic strengthening	ART adherence, viral suppression
Casalini C, 2023^44^	Controlled before-and-after study	Adolescents with HIV	N/A	N/A	Nigeria	General health facilities	Differentiated service delivery	Viral load testing coverage, viral suppression
Catania JA, 2021^45^	Randomized controlled trial	Vulnerable and orphaned adolescents	Older adolescents	49	United Republic of Tanzania (The)	Community and population-based	Digital interventions	Confirmatory testing and linkage-to-care
Chimbindi N, 2023^46^	Cohort study	Vulnerable and orphaned adolescents	Older adolescents	100	South Africa	Community and population-based	Digital interventions	Awareness of HIV infections
Cho H, 2019^47^	Cluster randomized controlled trial	Vulnerable and orphaned adolescents	Older adolescents	48	Kenya	School-based	School support	HIV infections
Cho H, 2018^48^	Cluster randomized controlled trial	Vulnerable and orphaned adolescents	Older adolescents	48.3	Kenya	School-based	School support	New HIV infections
Ciccaci F, 2023^49^	Controlled before-and-after study	Adolescents with HIV	N/A	N/A	Mozambique	General health facilities	Asset building	ART retention, rate of lost-to-follow-up
Delany-Moretlwe S, 2018^50^	Randomized controlled trial	Adolescents at risk of HIV	N/A	100	South Africa, Tanzania	N/A	Psychosocial support	PrEP continuation
Denison JA, 2022^51^	Randomized controlled trial	Adolescents with HIV	Older adolescents	62	Zambia	HIV/STD clinics	Multi-level intervention	ART adherence, viral failure
Dhakwa D, 2021^52^	Non-randomized controlled trial	Vulnerable and orphaned adolescents	Older adolescents	100	Zimbabwe	Community and population-based	M-Health	Services referral completion (reproductive health (RH) and HIV testing service (HTS))
Donenberg GR, 2022^53^	Randomized controlled trial	Adolescents with HIV	Older adolescents	51	Rwanda	HIV/STD clinics	Psychosocial support	ART Adherence, viral load
Dougherty G, 2022^54^	Interrupted time series study	Adolescents with HIV	N/A	N/A	Kenya	General health facilities	Clinical quality improvement	Repeat viral load test, viral suppression, adolescent on first-line ART whose regimens were switched
Dow DE, 2020^55^	Randomized controlled trial	Adolescents with HIV	Older adolescents	51	United Republic of Tanzania (The)	HIV/STD clinics	Mental health interventions	ART adherence, ART concentration in hair, viral load, viral suppression
Dow DE, 2022^56^	Randomized controlled trial	Adolescents with HIV	Older adolescents	53	United Republic of Tanzania (The)	HIV/STD clinics	Mental health interventions	ART adherence, viral load, viral suppression
Ekwunife OI, 2022^57^	Cluster randomized controlled trial	Adolescents with HIV	Early adolescents	50	Nigeria	HIV/STD clinics	Financial incentives	Proportion with undetectable viral load, CD4+ count, ART adherence, and retention in care
Floyd S, 2018^58^	Controlled before-and-after study	General adolescents	Older adolescents	59	Zambia	Community and population-based	Treatment as prevention	Awareness of HIV infections, ART initiation, retention in care
Galárraga O, 2020^59^	Non-randomized controlled trial	Perinatally adolescents living with HIV	Early adolescents	37.1	Ghana	HIV/STD clinics	Financial incentives	ART adherence, viral load, CD4+ count
Gitahi N, 2023^60^	Randomized controlled trial	Perinatally adolescents living with HIV	N/A	N/A	Kenya	N/A	Psychosocial support	ART adherence self-efficacy, viral load, viral suppression
Hallfors DD, 2015^61^	Cluster randomized controlled trial	Vulnerable and orphaned adolescents	Early adolescents	100	Zimbabwe	School-based	School support	HIV incidence
Hémono R, 2024^62^	Cluster randomized controlled trial	General adolescents	Older adolescents	51.5	Rwanda	School-based	Digital interventions	HIV testing
Hensen B, 2023^63^	Cluster randomized controlled trial	General adolescents	Older adolescents	50	Zambia	Community and population-based	Multi-level intervention	PrEP use, awareness of HIV infections, current use of ART
Hewett PC, 2016^64^	Cluster randomized controlled trial	Vulnerable and orphaned adolescents	N/A	100	Zambia	N/A	Asset building	HIV incidence
Hunter LA, 2020^65^	Randomized controlled trial	Vulnerable and orphaned adolescents	N/A	100	United Republic of Tanzania (The)	Drug shops	Differentiated service delivery	Distribution of HIVST kits, referrals for HIV services
Indravudh PP, 2019^66^	Cluster randomized controlled trial	General adolescents	Older adolescents	43.2	Malawi	Community and population-based	Self-care interventions	HIV testing, ART initiation
Jani N, 2016^67^	Controlled before-and-after study	Vulnerable and orphaned adolescents	Older adolescents	78.9	Ethiopia	Community and population-based	Psychosocial support	HIV testing
Jubilee M, 2019^68^	Non-randomized controlled trial	Adolescents with HIV	Older adolescents	53.1	Lesotho	Household and door-to-door	Targeted contact-based HIV testing interventions	HIV testing, HIV positivity rate, linked to care and treatment
Kabogo J, 2018^69^	Cohort study	Adolescents with HIV	N/A	48.6	Kenya	Community and population-based	Treatment as prevention	Viral suppression, optimal adherence to ART, suboptimal adherence to ART
Ketchaji A, 2019^70^	Randomized controlled trial	Adolescents with HIV	Older adolescents	N/A	Cameroon	General health facilities	Multi-level intervention	ART adherence, viral suppression
Kitetele FN, 2023^71^	Cohort study	Adolescents with HIV	N/A	58.2	Democratic Republic of the Congo	General health facilities	Disclosure support	Viral suppression, mortality rate
Kizito S, 2023^72^	Cluster randomized controlled trial	Adolescents with HIV	Early adolescents	56.4	Uganda	General health facilities	Economic strengthening	ART adherence, adherence self-efficacy
Kizito S, 2022^73^	Cluster randomized controlled trial	Adolescents with HIV	Early adolescents	56.3	Uganda	General health facilities	Economic strengthening	ART adherence
Kizito S, 2023^74^	Cluster randomized controlled trial	Adolescents with HIV	Early adolescents	58	Uganda	General health facilities	Economic strengthening	ART adherence
Kopo M, 2023^75^	Cluster randomized controlled trial	Adolescents with HIV	Older adolescents	71	Lesotho	General health facilities	Differentiated service delivery	Viral load, viral suppression, ART adherence, retention in care
Kose J, 2018^76^	Controlled before-and-after study	General adolescents	N/A	60.7	Kenya	General health facilities	Differentiated service delivery	HIV testing, HIV incidence, linked to care and treatment services
Kranzer K, 2018^77^	Randomized controlled trial	General adolescents	N/A	53.4	Zimbabwe	Household and door-to-door	Financial incentives	HIV testing
Kubheka SE, 2020^78^	Cohort study	Adolescents with HIV	N/A	45.4	South Africa	HIV/STD clinics	Clinical quality improvement	Viral suppression, viral load timeliness
Kuo C, 2020^79^	Randomized controlled trial	General adolescents	Early adolescents	56	South Africa	Community and population-based	Multi-level intervention	HIV testing
Letsela L, 2021^80^	Non-randomized cluster-controlled study	General adolescents	Early adolescents	50.3	South Africa	Household and door-to-door	Educational intervention	HIV testing, HIV incidence
Levy M, 2021^81^	Controlled before-and-after study	Pregnant and parenting adolescents	Older adolescents	100	Kenya	Household and door-to-door	Multi-level intervention	Retention in care, viral suppression
Linnemayr S, 2017^82^	Randomized controlled trial	Adolescents with HIV	Older adolescents	61	Uganda	HIV/STD clinics	M-Health	ART adherence
MacCarthy S, 2021^83^	Randomized controlled trial	Adolescents with HIV	Older adolescents	79.6	Uganda	HIV/STD clinics	M-Health	ART adherence
Mackenzie RK, 2017^84^	Cohort study	Adolescents with HIV	N/A	56.9	Malawi	HIV/STD clinics	Differentiated service delivery	ART initiation
Massa P, 2023^85^	Interrupted time series study	Transgender adolescent women	Older adolescents	0	Brazil	Online	Digital interventions	PrEP uptake, PrEP clinic scheduling
Mathur S, 2022^86^	Cohort study	Vulnerable and orphaned adolescents	Older adolescents	100	Kenya, Malawi, Zambia	Community and population-based	Asset building	HIV testing
Mavhu W, 2020^87^	Cluster randomized controlled trial	Adolescents with HIV	Older adolescents	52	Zimbabwe	General health facilities	Differentiated service delivery	Viral suppression, retention in care
Menna T, 2015^88^	Non-randomized controlled trial	General adolescents	Older adolescents	62.5	Ethiopia	School-based	Educational intervention	HIV testing
Merrill KG, 2023^89^	Randomized controlled trial	Vulnerable and orphaned adolescents	Older adolescents	100	South Africa	Community and population-based	Psychosocial support	HIV testing, PrEP uptake, PrEP adherence
Mthiyane N, 2022^90^	Cohort study	General adolescents	Older adolescents	100	South Africa	Community and population-based	Asset building	HIV testing and incidence, viral load
Muchabaiwa L, 2018^91^	Controlled before-and-after study	General adolescents	Older adolescents	56.3	Zimbabwe	Community and population-based	School support	HIV testing and prevalence
Mulwa S, 2021^92^	Cohort study	Vulnerable and orphaned adolescents	Early adolescents	100	Kenya	Community and population-based	Asset building	HIV testing and counseling
Munyayi FK, 2020^93^	Cohort study	Adolescents with HIV	Early adolescents	59	Namibia	HIV/STD clinics	Differentiated service delivery	ART retention, ART regimen
Munyayi FK, 2020^94^	Cohort study	Adolescents with HIV	Early adolescents	59	Namibia	HIV/STD clinics	Differentiated service delivery	Adherence to ART, Virologic suppression, ART regimen
Musanje K, 2024^95^	Randomized controlled trial	Adolescents with HIV	Older adolescent	59	Uganda	General health facilities	Mental health interventions	ART adherence
Nabunya P, 2024^96^	Cluster randomized controlled trial	Adolescents with HIV	Early adolescents	62.9	Uganda	HIV/STD clinics	Psychosocial support	ART adherence
Nair G, 2023^97^	Randomized controlled trial	Vulnerable and orphaned adolescents	Older adolescents	100	South Africa, Zimbabwe, Uganda	General health facilities	Novel prevention options	PrEP adherence, dapivirine vaginal ring adherence
Ness TE, 2021^98^	Controlled before-and-after study	Adolescents with HIV	Older adolescents	30	Eswatini	General health facilities	Psychosocial support	Viral load, CD4 counts
Nwanja E, 2023^99^	Historically controlled study	Pregnant and parenting adolescents	Older adolescents	100	Nigeria	Mixed settings	Prevention of mother-to-child transmission	HIV testing, HIV positivity rate
Oberth G, 2021^100^	Historically controlled study	Vulnerable and orphaned adolescents	Early adolescents	100	Zimbabwe	Household and door-to-door	School support	HIV testing
Olashore AA, 2023^101^	Randomized controlled trial	Adolescents with HIV	Older adolescents	76	Botswana	General health facilities	Mental health interventions	ART adherence
Osita EE, 2022^102^	Randomized controlled trial	Adolescents at risk of HIV	Older adolescents	N/A	Nigeria	School-based	Mental health interventions	HIV voluntary counseling and testing
Patel RC, 2022^103^	Randomized controlled trial	Adolescents with HIV	Early adolescents	49	Kenya	General health facilities	Point-of-care viral load testing and targeted drug resistance mutation testing	Viral suppression
Pettifor A, 2016^104^	Randomized controlled trial	Adolescents at risk of HIV	Older adolescents	100	South Africa	School-based	Financial incentives	HIV incidence
Phiri MM, 2024^105^	Cluster randomized controlled trial	General adolescents	Older adolescents	55.5	Zambia	Community and population-based	Multi-level intervention	HIV testing
Pike C, 2023^106^	Cluster randomized controlled trial	Vulnerable and orphaned adolescents	Older adolescents	100	South Africa	School-based	Educational intervention	HIV testing, HIV incidence
Rucinski K, 2022^107^	Cohort study	Adolescents at risk of HIV	Older adolescents	100	Malawi	Community and population-based	Differentiated service delivery	HIV testing, HIV incidence, ART initiation
Ruria EC, 2017^108^	Controlled before-and-after study	Adolescents at risk of HIV	Older adolescents	86	Kenya	Mixed settings	Psychosocial support	Linkage to services and retention in care, ART initiation
Sakthivel R, 2023^109^	Randomized controlled trial	Adolescents with HIV	Early adolescents	49	India	HIV/STD clinics	Psychosocial support	ART adherence, level of CD4 count
Shanaube K, 2021^110^	Cluster randomized controlled trial	General adolescents	N/A	75.9	Zambia, South Africa	Household and door-to-door	Treatment as prevention	Uptake of testing, ART coverage and time to initiate ART, Retention on ART, HIV treatment and care cascade
Shanaube K, 2017^111^	Cluster randomized controlled trial	General adolescents	Older adolescents	50.3	Zambia	Household and door-to-door	Treatment as prevention	HIV counseling and testing uptake, proportion of HIV positives
Simms V, 2022^112^	Cluster randomized controlled trial	Adolescents with HIV	Early adolescents	55.5	Zimbabwe	General health facilities	Mental health interventions	Viral load, virological non-suppression
Speizer IS, 2020^113^	Cluster randomized controlled trial	General adolescents	Early adolescents	100	South Africa	School-based	School support	HIV testing, HIV positive
Speizer IS, 2020^114^	Cluster randomized controlled trial	General adolescents	Older adolescents	55.1	South Africa	School-based	School support	HIV prevalence
Ssewamala FM, 2020^115^	Cluster randomized controlled trial	Adolescents with HIV	Early adolescents	55.21	Uganda	General health facilities	Economic strengthening	VL suppression, ART medications
Stangl AL, 2021^116^	Controlled before-and-after study	Adolescents with HIV	Older adolescents	100	Zambia	General health facilities	Psychosocial support	ART adherence
Thurman TR, 2024^117^	Controlled before-and-after study	Adolescents with HIV	Older adolescents	65.7	South Africa	Community and population-based	Psychosocial support	ART initiation, ART adherence
Tozan Y, 2021^118^	Cluster randomized controlled trial	Adolescents with HIV	Early adolescents	56.41	Uganda	General health facilities	Economic strengthening	Viral suppression
Trapence CP, 2023^119^	Cohort study	Adolescents with HIV	Older adolescents	59	Malawi	HIV/STD clinics	Differentiated service delivery	ART initiation, retention in care
Tunje A, 2024^120^	Non-randomized controlled trial	Adolescents with HIV	Older adolescents	43.8	Ethiopia	HIV/STD clinics	M-Health	ART adherence, retention in care
Tymejczyk O, 2020^121^	Interrupted time series study	Adolescents with HIV	Early adolescents	58.3	Burundi, Democratic Republic of the Congo, Kenya, Malawi, Rwanda, Uganda, Zambia	General health facilities	Treatment as prevention	Rapid ART Initiation
Vreeman RC, 2019^122^	Cluster randomized controlled trial	Adolescents with HIV	Early adolescents	52.6	Kenya	General health facilities	Differentiated service delivery	Viral load suppression, CD4
Waidler J, 2022^123^	Cluster randomized controlled trial	General adolescents	Older adolescents	46	United Republic of Tanzania (The)	Community and population-based	Multi-level intervention	HIV testing
Wango GN, 2023^124^	Randomized controlled trial	Adolescents at risk of HIV	Older adolescents	100	Kenya	Community and population-based	Self-care interventions	HIV testing, tested HIV positive
Willis N, 2019^125^	Randomized controlled trial	Adolescents with HIV	Early adolescents	60.6	Zimbabwe	Community and population-based	Differentiated service delivery	Linkage to services and retention in care, adherence to ART
Wirsiy FS, 2022^126^	Randomized controlled trial	General adolescents	Older adolescents	100	Cameroon	Household and door-to-door	M-Health	HIV testing practices
Yumo HA, 2018^127^	Non-randomized controlled trial	General adolescents	N/A	N/A	Cameroon	General health facilities	Treatment as prevention	HIV testing, HIV case detection/positivity rate, linkage, and ART enrolment
Zanoni BC, 2024^128^	Randomized controlled trial	Adolescents with HIV	Older adolescents	46.3	South Africa	General health facilities	M-Health	Retention in care, viral suppression
Zulaika G, 2023^129^	Cluster randomized controlled trial	Vulnerable and orphaned adolescents	Older adolescents	100	Kenya	School-based	Financial incentives	HIV incidence

**Table 2:** Included study characteristics (n = 99, 441,252 participants).

	Number of studies (%)	Number of participants
**Study design**		
Randomized controlled trial	27 (27.3%)	10,376
Cluster randomized controlled trial	30 (30.3%)	171,337
Non-randomized controlled trial	11 (11.1%)	22,483
Cohort study	14 (14.1%)	14,406
Controlled before-and-after study	11 (11.1%)	109,441
Interrupted time series study	3 (3.0%)	7423
Historically controlled study	2 (2.0%)	101,968
Non-randomized cluster-controlled study	1 (1.0%)	3818
**Population**		
Adolescents with HIV	46 (46.5%)	28,413
General adolescents living in high HIV-burden contexts	22 (22.2%)	266,769
Vulnerable and orphaned adolescents	19 (19.2%)	130,226
Adolescents at risk of HIV	5 (5.1%)	3726
Perinatally adolescents living with HIV	4 (4.0%)	889
Pregnant and parenting adolescents	2 (2.0%)	10,740
Transgender adolescent women	1 (1.0%)	489
**Age**		
Older adolescents (15–19)	56 (56.6%)	136,390
Early adolescents (10–14)	26 (26.3%)	114,508
Not specified	17 (17.2%)	190,354
**Gender**		
Girl	25 (25.3%)	147,093
Boy	1 (1.1%)	489
Mixed gender	65 (65.7%)	282,377
Not specified	8 (8.1%)	11,293
**Study setting**		
Community and population-based	26 (26.3%)	86,276
General health facilities	25 (25.3%)	102,046
HIV/STD clinics	21 (21.2%)	6489
School-based	11 (11.1%)	30,508
Household and door-to-door	8 (8.1%)	197,958
Online	2 (2.0%)	1568
Mixed settings	2 (2.0%)	10,633
Drug shops	1 (1.0%)	20
Not specified	3 (3.0%)	5754
**Coverage/Scope of the study**		
Global	0 (0)	0
Multi-countries	7 (7.1%)	99,406
National	3 (3.0%)	105,677
Local	89 (89.9%)	236,169
**Number of sites**		
Multi-site	78 (78.8%)	428,323
Single site	19 (19.2%)	5731
Not specified	2 (2.0%)	7198
**UNICEF region**		
Eastern and Southern Africa (ESARO)	82 (82.8%)	405,985
West and Central Africa (WCARO)	13 (13.1%)	26,741
Latin America and Caribbean (TACRO)	2 (2.0%)	1226
South Asia (ROSA)	1 (1.0%)	388
Mixed regions	1 (1.0%)	6912
**Country income status**		
Low income	23 (23.2%)	19,980
Lower-middle income	51 (51.5%)	288,115
Upper-middle income	18 (18.2%)	33,751
High income	0 (0)	0
Mixed-income	7 (7.1%)	99,406
**Intervention types**		
Differentiated service delivery	15 (15.2%)	89,313
Psychosocial support	12 (12.1%)	3036
Multi-level interventions	9 (9.1%)	33,448
Economic strengthening	7 (7.1%)	4500
Educational intervention	7 (7.1%)	121,315
M-Health	7 (7.1%)	10,194
Mental health interventions	6 (6.1%)	1332
Asset building	6 (6.1%)	13,209
Treatment as prevention	5 (5.1%)	120,916
Financial incentives	5 (5.1%)	8301
Digital Interventions	5 (5.1%)	10,087
Self-care interventions	3 (3.0%)	4807
School support	3 (3.0%)	1998
Disclosure support	2 (2.0%)	1539
Clinical quality improvement	2 (2.0%)	522
Targeted contact-based HIV testing interventions	2 (2.0%)	5807
Novel prevention options	1 (1.0%)	247
Point-of-care viral load testing and targeted drug resistance mutation testing	1 (1.0%)	325
Prevention of mother-to-child transmission	1 (1.0%)	10,356

Of the 99 studies, the risk of bias was rated as high in 37 of the studies (37.4%), medium in 31 (31.1%), and low in 31 studies (31.1%; Appendix pp 13–15). Overall, the quality of evidence of HIV care cascade-related outcomes was graded as being moderate to low due to the considerable heterogeneity across studies and limited included studies (Table 3).

**Table 3:** Grading the quality of evidence for HIV prevention and care cascade data.

Outcome	Evidence	Risk of bias	Inconsistency	Imprecision	Indirectness	QOE	Main findings
**Educational interventions**							
HIV testing	Two observational studies (N = 2534) One randomized controlled trial (N = 2954)	No serious risk of bias (Three observation studies with low risk of bias)	Serious inconsistency (I^2^: 86.0%)	No imprecision	No indirectness	Moderate	Relative risk: 1.30 (95% confidence interval: 1.04–1.62)
**Differentiated service delivery**							
ART adherence	One observational study (N = 385) One randomized controlled trial (N = 75)	Serious risk of bias (one RCT with some risk of bias, one observational study with medium risk of bias)	Serious inconsistency (I^2^: 76.5%)	Serious imprecision^a^	No indirectness	Low	Relative risk: 1.29 (95% confidence interval: 0.78–2.14)
ART retention	Four observational studies (N = 1691) One randomized controlled trial (N = 479)	No serious risk of bias (one RCT with low risk of bias, one observational study with high risk of bias, two observational studies with medium risk of bias, one observational study with low risk of bias)	Serious inconsistency (I^2^: 87.6%)	No imprecision	No indirectness	Moderate	Relative risk: 1.18 (95% confidence interval: 1.04–1.36)
Virological suppression	Five observational studies (N = 4680) Two randomized controlled trials (N = 647)	No serious risk of bias (one RCT with some risk of bias, one RCT with low risk of bias, two observational studies with high risk of bias, one observational study with medium risk of bias, two observational studies with low risk of bias)	Serious inconsistency (I^2^: 92.5%)	No imprecision	No indirectness	Moderate	Relative risk: 1.19 (95% confidence interval: 1.06–1.33)
**Multi-level interventions**							
PrEP uptake	One randomized controlled trial (N = 1747)	Very serious risk of bias (one RCT with a high risk of bias)	No inconsistency	Serious imprecision^a^	No indirectness	Low	Relative risk: 1.18 (95% confidence interval: 0.27–5.28)
HIV testing	One observational study (N = 12,925) Three randomized controlled trials (N = 24,697)	No serious risk of bias (one small RCT with a high risk of bias, one RCT with some risk of bias, one RCT with low risk of bias, one observational study with a high risk of bias)	Serious inconsistency (I^2^: 96.4%)	No imprecision	No indirectness	Moderate	Relative risk: 6.70 (95% confidence interval: 3.98–11.27)
Awareness of HIV infection	One randomized controlled trial (N = 1000)	Very serious risk of bias (one RCT with a high risk of bias)	No inconsistency	No imprecision	No indirectness	Moderate	Relative risk: 1.84 (95% confidence interval: 1.31–2.57)
Virological suppression	One observational study (N = 32) One randomized controlled trial (N = 49)	Serious risk of bias (one small RCT with a high risk of bias, one small observational study with a low risk of bias)	Serious inconsistency (I^2^: 87.4%)	Serious imprecision^a^	No indirectness	Low	Relative risk: 1.63 (95% confidence interval: 0.46–5.78)
**Mental health interventions**							
Virological suppression	Three randomized controlled trials (N = 922)	No serious risk of bias (three RCTs with low risk of bias)	No inconsistency (I^2^: 0)	Serious imprecision^a^	No indirectness	Moderate	Relative risk: 0.97 (95% confidence interval: 0.92–1.03)
**Economic Strengthening**							
ART adherence	One randomized controlled trial (N = 767)	Very serious risk of bias (one RCT with a high risk of bias)	No inconsistency	No imprecision	No indirectness	Moderate	Relative risk: 1.06 (95% confidence interval: 1.00–1.12)
Virological suppression	Three randomized controlled trials (N = 1959)	No serious risk of bias (two RCTs with some risk of bias, one RCT with low risk of bias)	No inconsistency (I^2^: 0)	Serious imprecision^a^	No indirectness	Moderate	Relative risk: 1.04 (95% confidence interval: 0.98–1.11)
**Treatment as prevention**							
HIV testing	One randomized controlled trial (N = 88,137)	No serious risk of bias (one large RCT with low risk of bias)	No inconsistency	Serious imprecision^a^	No indirectness	Moderate	Relative risk: 1.00 (95% confidence interval: 0.99–1.01)
Awareness of HIV infection	One observational study (N = 28,746) Two randomized controlled trials (N = 108,472)	Serious risk of bias (one RCT with a high risk of bias, one RCT with a low risk of bias, one observational study with high risk of bias)	Serious inconsistency (I^2^: 99.6%)	No imprecision	No indirectness	Moderate	Relative risk: 2.96 (95% confidence interval: 2.48–3.54)
ART adherence	One observational study (N = 205)	Serious risk of bias (one small observational study with medium risk of bias)	No inconsistency	Serious imprecision^a^	No indirectness	Low	Relative risk: 1.05 (95% confidence interval: 0.65–1.70)
Virological suppression	One observational study (N = 205)	Serious risk of bias (one small observational study with medium risk of bias)	No inconsistency	Serious imprecision^a^	No indirectness	Low	Relative risk: 0.98 (95% confidence interval: 0.83–1.14)
**Asset building**							
HIV testing	Two observational studies (N = 3486) One randomized controlled trial (N = 2851)	Serious risk of bias (two observational studies with moderate risk of bias, one RCT with a low risk of bias)	Serious inconsistency (I^2^: 95.9%)	No imprecision	No indirectness	Low	Relative risk: 1.38 (95% confidence interval: 1.15–1.65)
Awareness of HIV infection	One randomized controlled trial (N = 1690)	Serious risk of bias (one RCT with moderate risk of bias)	No inconsistency	No imprecision	No indirectness	Moderate	Relative risk: 1.54 (95% confidence interval: 1.31–1.81)
ART retention	One observational study (N = 738)	No serious risk of bias (one observational study with low risk of bias)	No inconsistency	No imprecision	No indirectness	Moderate	Relative risk: 1.05 (95% confidence interval: 1.02–1.08)
Virological suppression	One observational study (N = 1895)	Serious risk of bias (one observational study with medium risk of bias)	No inconsistency	Serious imprecision^a^	No indirectness	Low	Relative risk: 1.02 (95% confidence interval: 0.99 to 1.05)
**Self-care interventions**							
HIV testing	Three randomized controlled trials (N = 4782)	Serious risk of bias (two RCTs with high risk of bias, one smaller RCT with low risk of bias)	Serious inconsistency (I^2^: 98.0%)	No imprecision	No indirectness	Low	Relative risk: 1.45 (95% confidence interval: 1.07 to 1.97)
Awareness of HIV infection	One randomized controlled trial (N = 2681)	Serious risk of bias (one RCT with a high risk of bias)	No inconsistency	Serious imprecision^a^	No indirectness	Low	Relative risk: 1.02 (95% confidence interval: 0.84–1.24)
**M-health**							
HIV testing	One observational study (N = 3486) One randomized controlled trial (N = 398)	Serious risk of bias (One smaller RCT with low risk of bias, one observational study with high risk of bias)	Moderate inconsistency (I^2^: 53.1%)	Serious imprecision^a^	No indirectness	Low	Relative risk: 1.12 (95% confidence interval: 0.84–1.48)
ART adherence	One observational study (N = 303) Three randomized controlled trials (N = 733)	No serious risk of bias (one RCT with high risk of bias, one RCT with some risk of bias, one RCT with low risk of bias, one observational study with some risk of bias)	Serious inconsistency (I^2^: 79.1%)	Serious imprecision^a^	No indirectness	Moderate	Relative risk: 1.06 (95% confidence interval: 0.86–1.31)
ART retention	One randomized controlled trial (N = 80)	Very serious risk of bias (one small RCT with a high risk of bias)	No inconsistency	Serious imprecision^a^	No indirectness	Low	Relative risk: 1.11 (95% confidence interval: 0.98–1.27)
Virological suppression	One randomized controlled trial (N = 209)	No serious risk of bias (one RCT with some risk of bias)	No inconsistency	No imprecision	No indirectness	Moderate	Relative risk: 1.36 (95% confidence interval: 1.04–1.77)
**Financial incentives**							
HIV testing	One randomized controlled trial (N = 2160)	Serious risk of bias (one RCT with moderate risk of bias)	No inconsistency	No imprecision	No indirectness	Moderate	Relative risk: 2.23 (95% confidence interval: 1.84–2.70)
ART adherence	One observational Study (N = 70) One randomized controlled trial (N = 246)	No serious risk of bias (one RCT with low risk of bias, one observational study with low risk of bias)	No inconsistency (I^2^: 0)	No imprecision	No indirectness	Moderate	Relative risk: 1.52 (95% confidence interval: 1.23–1.89)
ART retention	One randomized controlled trial (N = 246)	No serious risk of bias (one RCT with low risk of bias)	No inconsistency	Serious imprecision^a^	No indirectness	Moderate	Relative risk: 1.03 (95% confidence interval: 0.91–1.16)
Virological suppression	One observational Study (N = 70) One randomized controlled trial (N = 246)	No serious risk of bias (one RCT with low risk of bias, one small observational study with medium risk of bias)	Low inconsistency (I^2^: 18.6%)	Serious imprecision^a^	No indirectness	Moderate	Relative risk: 0.88 (95% confidence interval: 0.48, 1.62)
**Psychosocial support**							
PrEP uptake	One randomized controlled trial (N = 37)	Very serious risk of bias (one small RCT with high risk of bias)	No inconsistency	Serious imprecision^a^	No indirectness	Low	Relative risk: 1.61 (95% confidence interval: 0.99 to 2.61)
HIV testing	One observational study (N = 834) One randomized controlled trial (N = 49)	Serious risk of bias (one small RCT with low risk of bias, one observational study with high risk of bias)	Serious inconsistency (I^2^: 89.5%)	Serious imprecision^a^	No indirectness	Low	Relative risk: 1.23 (95% confidence interval: 0.87–1.74)
ART adherence	Two observational Studies (N = 828)	Serious risk of bias (one observational study with medium risk of bias, one observational study with low risk of bias)	No inconsistency (I^2^: 0)	No imprecision	No indirectness	Moderate	Relative risk: 1.20 (95% confidence interval: 1.07–1.36)
Virological suppression	One observational Study (N = 64)	Serious risk of bias (one small observational study with medium risk of bias)	No inconsistency	Serious imprecision^a^	No indirectness	Low	Relative risk: 1.08 (95% confidence interval: 0.70–1.66)
**Digital interventions**							
PrEP uptake	One observational study (N = 619)	No serious risk of bias (one observational study with low risk of bias)	No inconsistency	Serious imprecision^a^	No indirectness	Low	Relative risk: 2.02 (95% confidence interval: 0.99 to 4.09)
HIV testing	One observational study (N = 5378) One randomized controlled trial (N = 9255)	Serious risk of bias (one RCT with a high risk of bias, one observational study with a high risk of bias)	Serious inconsistency (I^2^: 98.4%)	No imprecision	No indirectness	Low	Relative risk: 1.79 (95% confidence interval: 1.23–2.61)
Awareness of HIV infection	Two observational Studies (N = 7, 248)	Serious risk of bias (one observational study with high risk of bias, one observational study with medium risk of bias)	Serious inconsistency (I^2^: 99.0%)	Serious imprecision^a^	No indirectness	Low	Relative risk: 1.64 (95% confidence interval: 1.00–2.68)
**Disclosure support**							
Virological suppression	Two observational Studies (N = 215)	Very serious risk of bias (two small observational studies with high risk of bias)	No inconsistency (I^2^: 0)	No imprecision	No indirectness	Moderate	Relative risk: 1.89 (95% confidence interval: 1.39–2.58)
**Clinical quality improvement**							
Virological suppression	Two observational Studies (N = 2252)	Serious risk of bias (two observational studies with high risk of bias)	Serious inconsistency (I^2^: 99.2%)	Serious imprecision^a^	No indirectness	Low	Relative risk: 1.26 (95% confidence interval: 0.74–2.14)
**Targeted contact-based HIV testing**							
HIV testing	One observational study (N = 4719)	Serious risk of bias (one observational study with a high risk of bias)	No inconsistency	No imprecision	No indirectness	Moderate	Relative risk: 0.63 (95% confidence interval: 0.60–0.65)
**Point-of-care viral load testing and targeted drug resistance mutation testing**							
Virological suppression	One randomized controlled trial (N = 243)	No serious risk of bias (one RCT with some risk of bias)	No inconsistency	Serious imprecision^a^	No indirectness	Moderate	Relative risk: 0.95 (95% confidence interval: 0.89–1.02)

a 95% confidence intervals crosses 1.

Publication bias was also assessed for each HIV care cascaded outcome (Appendix pp 18–23). Specifically, funnel plots (Appendix p 19) and Egger's test (p = 0.002) indicated a publication bias in the intervention studies on HIV testing.

**Fig. 2:**
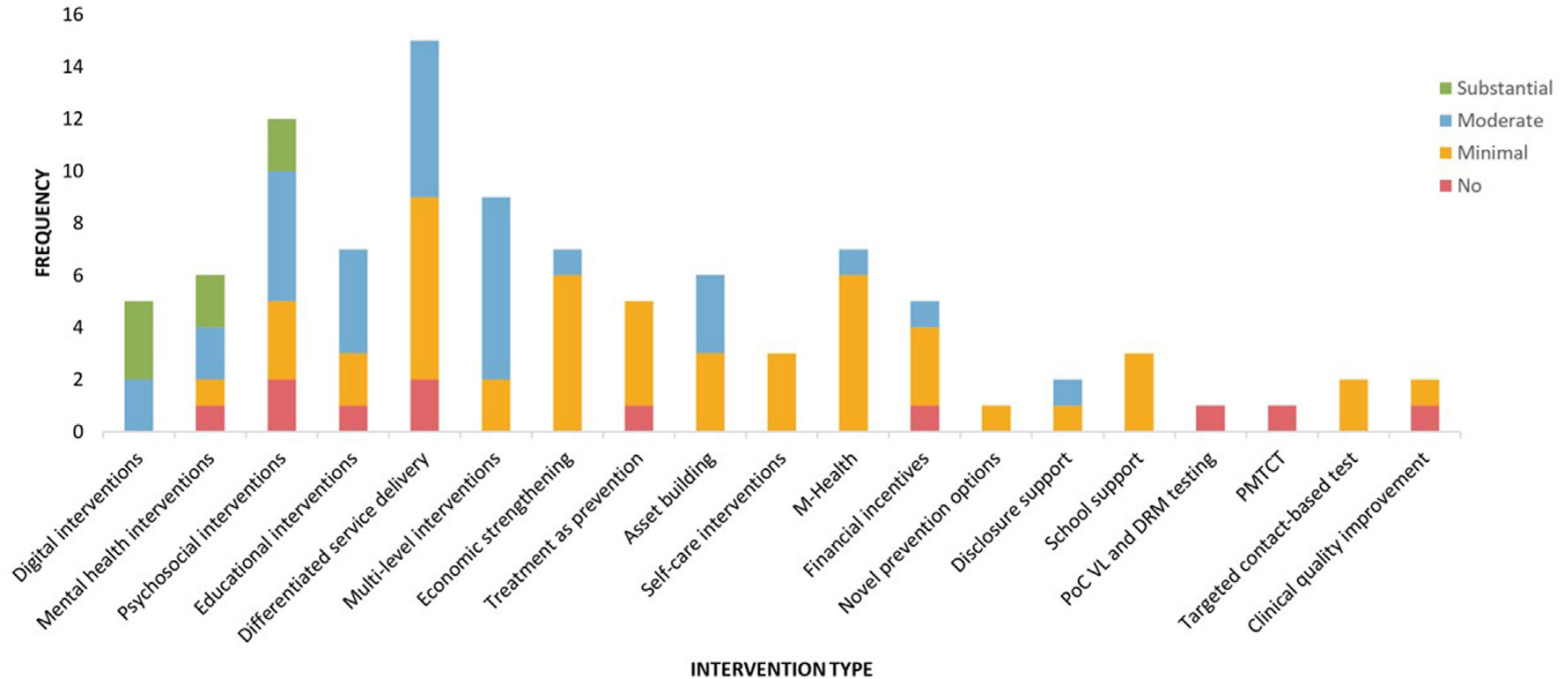
The overall degree of adolescent engagement in different HIV prevention and care interventions.

Most of the identified studies demonstrated minimal to moderate levels of adolescent engagement throughout the research activities from start to finish, as determined by our assessment using Hart's Ladder assessment tool (Fig. 2). Of these, 6 had substantial adolescent engagement, 33 had moderate, 48 had minimal, and 12 had none. Substantial adolescent engagement was primarily seen in the study design and implementation of digital interventions (60.0%), mental health interventions (33.3%), and psychosocial support (16.7%). Additionally, multi-level intervention (77.8%), educational interventions (57.1%), disclosure support (50.0%), and asset building (50.0%) documented relatively high levels of moderate adolescent engagement. In contrast, self-care interventions (100.0%), school support (100.0%), novel prevention options (100.0%), and targeted contact-based HIV testing interventions (100.0%) appear to have minimum adolescent engagement in their design and delivery.

### HIV prevention-to-care cascade

Table 4 summarizes the key interventions assessed and their effects across different stages of the cascade. There was substantial heterogeneity across the studies. Table 4 and the figures (Appendix p 17, Fig. 3, Fig. 4, Fig. 5, Fig. 6, Fig. 7) include the I^2^ statistic to indicate heterogeneity levels.

**Table 4:** Effect of different interventions on the outcomes across the HIV prevention and care cascade.

**Fig. 3:**
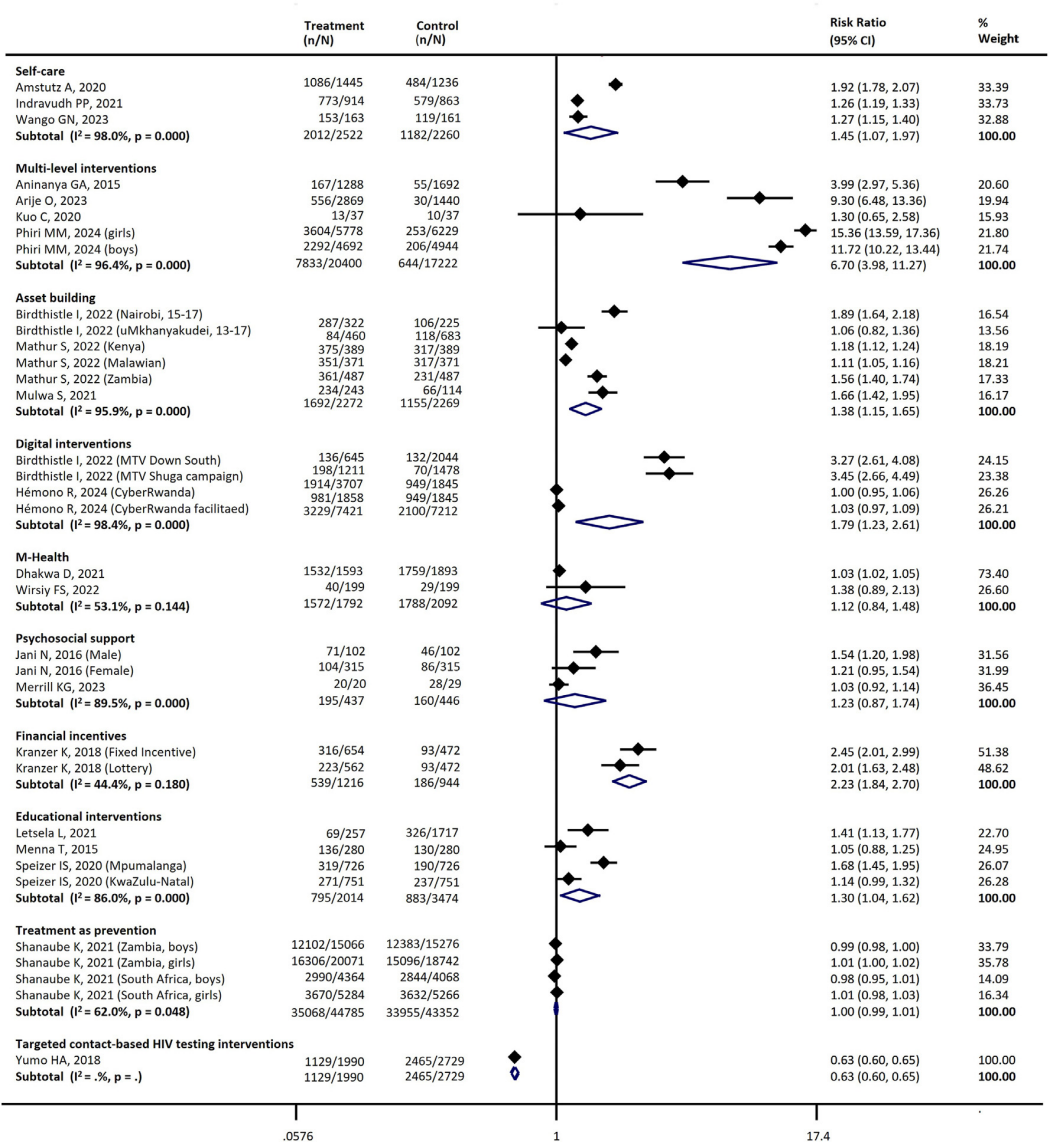
Forest plot examining the association between interventions and HIV testing.

**Fig. 4:**
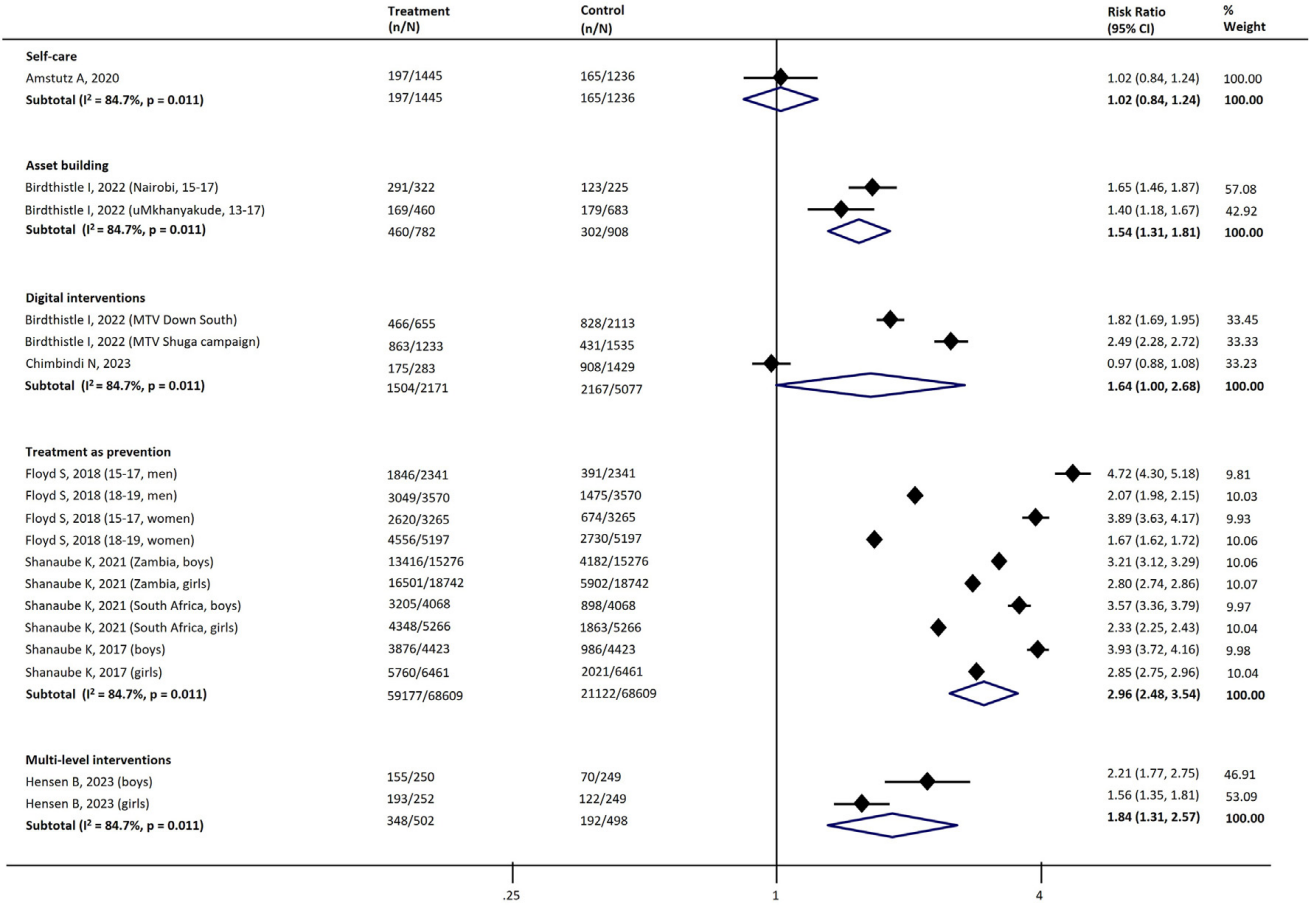
Forest plot examining the association between interventions and awareness of HIV-infected status.

**Fig. 5:**
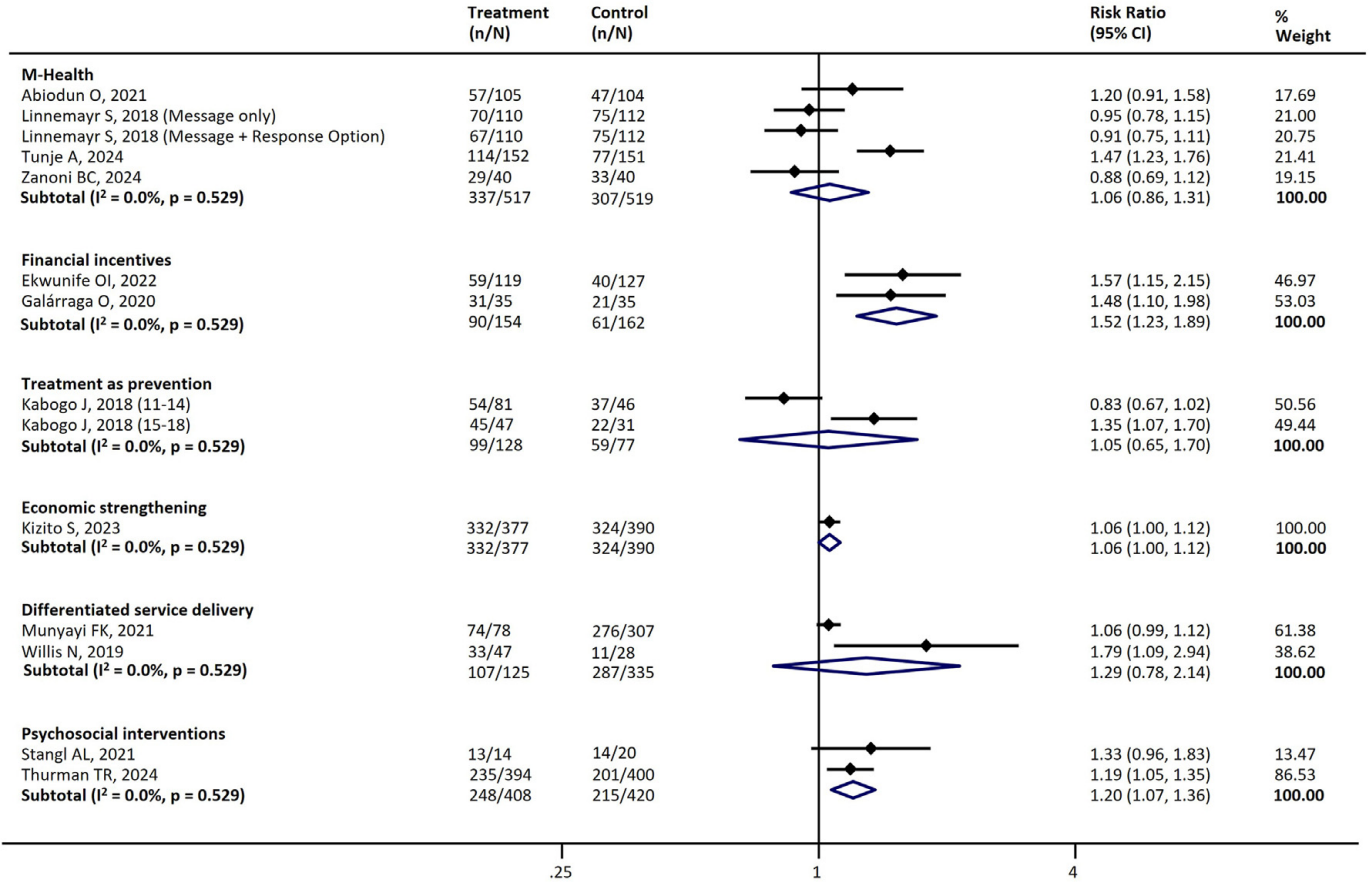
Forest plot examining the association between interventions and ART adherence.

**Fig. 6:**
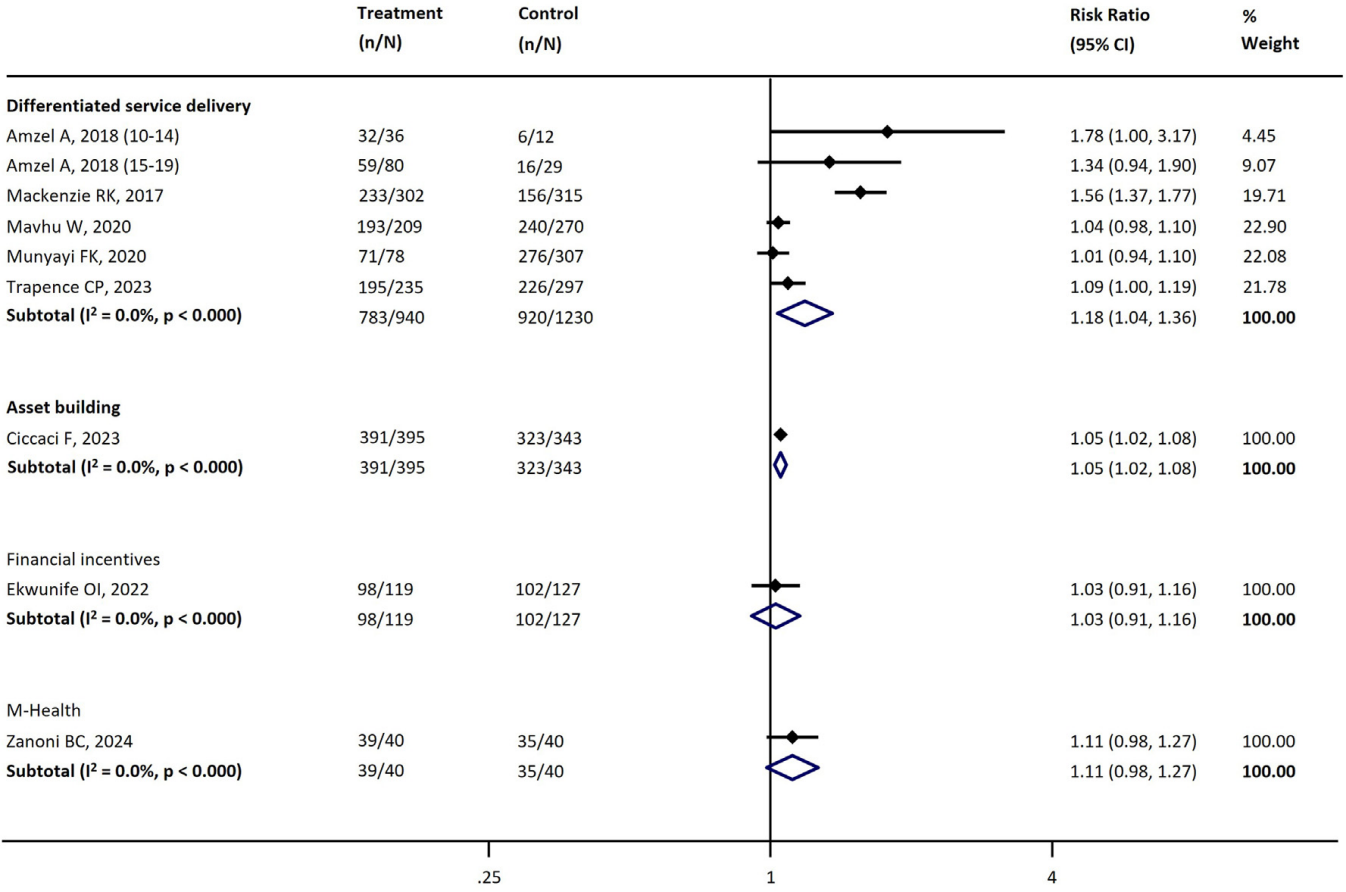
Forest plot examining the association between interventions and ART retention.

**Fig. 7:**
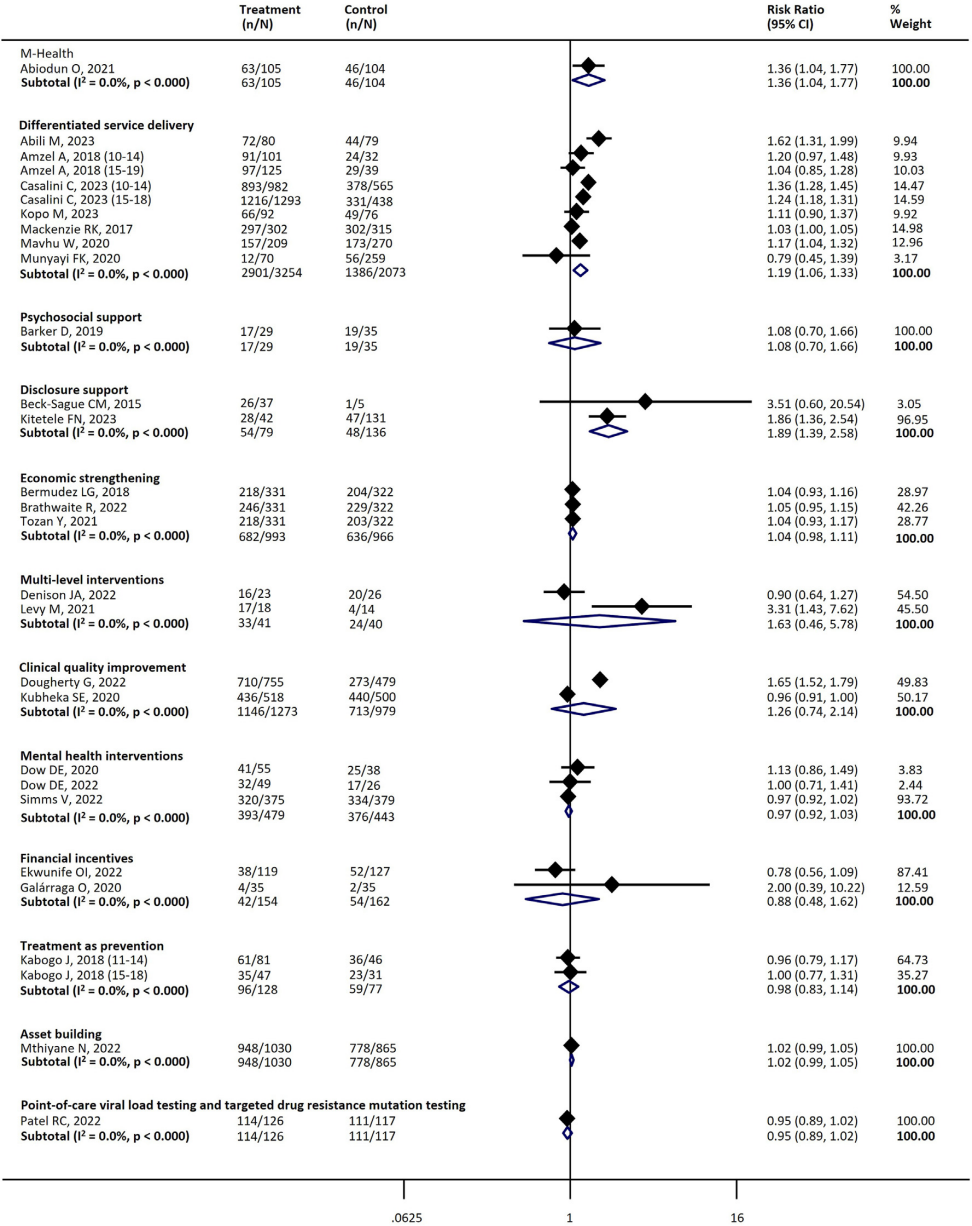
Forest plot examining the association between interventions and virological suppression.

### ARV-based prevention

Three intervention types were assessed for their effect on PrEP uptake.^63^^,^^85^^,^^89^ Digital interventions (two study arms from one study, RR: 2.02, 95% CI 0.99–4.09) showed a potential effect. A multi-level intervention (one study, RR: 1.18, 95% CI 0.27–5.28) demonstrated a similar effect. Similarly, psychosocial support (one study, RR: 1.61, 95% CI 0.99–2.61) produced comparable results (Table 4, Appendix p 17).

#### Testing

Ten types of interventions with 22 studies were assessed for their impact on HIV testing.^33^^,^^35^^,^^36^^,^^41^^,^^42^^,^^52^^,^^62^^,^^66^^,^^67^^,^^77^^,^^79^^,^^80^^,^^86^^,^^88^^,^^89^^,^^92^^,^^105^^,^^110^^,^^113^^,^^124^^,^^126^^,^^127^ The following six interventions were associated with improved HIV testing uptake compared with a comparator or control when data were pooled: educational interventions (Three studies; RR: 1.30, 95% CI 1.04–1.62); digital intervention (two studies; RR: 1.79, 95% CI 1.23–2.61); multi-level intervention (four studies; RR: 6.70, 95% CI 3.98–11.27); financial incentives (one study; RR: 2.23, 95% CI 1.84–2.70); self-care (three studies; RR 1.45, 95% CI 1.07–1.97); asset building (three studies; RR: 1.38, 95% CI 1.15–1.65) (Table 4, Fig. 3).

#### Awareness of HIV infections

Five types of interventions with eight studies were assessed for their impact on people's awareness of their HIV infections.^33^^,^^41^^,^^42^^,^^46^^,^^58^^,^^63^^,^^110^^,^^111^ The following four interventions significantly improved adolescent's awareness of HIV infections compared with a comparator/control when data were pooled: treatment as prevention (Three studies; RR 2.96, 95% CI 2.48–3.54); multi-level intervention (one study; RR 1.84, 95% CI 1.31–2.57) and asset building (one study; RR: 1.54, 95% CI 1.31–1.81) (Table 4, Fig. 4).

#### Adherence

Six types of interventions with 12 studies were assessed for their impact on ART adherence.^31^^,^^57^^,^^59^^,^^69^^,^^72^^,^^82^^,^^93^^,^^116^^,^^117^^,^^120^^,^^125^^,^^128^ Financial incentives (two studies; RR 1.52, 95% CI 1.23–1.89) and psychosocial support (two studies; RR 1.20, 95% CI 1.07–1.36) significantly improved adherence, while economic strengthening may improve adherence (one study; RR 1.06, 95% CI 1.00–1.12) (Table 4, Fig. 5).

#### ART retention

Four types of interventions with eight studies were assessed for their impact on ART retention.^34^^,^^49^^,^^57^^,^^84^^,^^87^^,^^93^^,^^119^^,^^128^ Notably, differentiated service delivery interventions (five studies; RR 1.18, 95% CI 1.04–1.36) and asset building (one study; RR 1.05, 95% CI 1.02–1.08) were associated with increased ART retention compared to a comparator (Table 4, Fig. 6).

#### Viral suppression

Twelve types of interventions with 26 studies were assessed for their impact on virological suppression.^31^^,^^32^^,^^34^^,^^37^^,^^39^^,^^40^^,^^43^^,^^44^^,^^51^^,^^54^, ^55^, ^56^, ^57^^,^^59^^,^^69^^,^^71^^,^^75^^,^^78^^,^^81^^,^^84^^,^^87^^,^^90^^,^^94^^,^^103^^,^^112^^,^^118^ The following three interventions significantly increased the proportion of adolescents with virological suppression when data were pooled compared with a comparator: m-health (one study; RR 1.36, 95% CI 1.04–1.77); differentiated service delivery (seven studies; RR 1.19, 95% CI 1.06–1.33), disclosure support (two studies; RR 1.89, 95% CI 1.39–2.58) (Table 4, Fig. 7).

Adolescents of different levels of engagement also exhibit effects on the outcomes across the HIV prevention and care continuum. Specifically, all three studies on PrEP-related interventions demonstrated moderate adolescent engagement, with a combined RR of 1.82 (95% CI: 1.24–2.66). For HIV testing, moderate to substantial adolescent engagement yielded an RR of 2.37 (95% CI: 1.43–3.93; nine studies), compared to an RR of 1.23 (95% CI: 1.15–1.31; 13 studies) for minimum adolescent engagement (Appendix pp 16).

### Secondary outcomes

A total of 29 studies examined the effect of HIV interventions on related mental, social and behavioral, and sexual health outcomes. These included mental health (five studies), exposure to violence (four studies), school drop-out (six studies), HIV stigma (five studies), transactional sex (three studies), condom use (nine studies), adolescent pregnancy (seven studies), STI co-infection (eight studies), and utilization of sexual and reproductive health services (seven studies).

### Mental health

Two types of interventions with five studies were assessed for their impact on adolescents' mental health.^55^^,^^56^^,^^87^^,^^112^^,^^122^ Notably, mental health interventions (three studies; SMD −0.29, 95% CI −0.42 to 0.16) significantly decreased symptoms of mental ill health among adolescents compared with a comparator/standard of care (Table 5, Appendix p 24).

**Table 5:** Effect of different interventions on mental health-related outcomes.

#### Exposure to violence

Three types of interventions (economic strengthening, asset building, and self-care interventions) with four studies were assessed for their impact on adolescents' exposure to violence.^86^^,^^92^^,^^104^^,^^124^ Among them, asset building (two studies; RR 0.75, 95% CI 0.59–0.97) and economic strengthening (one study; RR 0.64, 95% CI 0.58–0.71) significantly reduced the proportion of violence exposure among adolescents compared with a comparator (Table 6, Appendix p 25).

**Table 6:** Effect of interventions on selected social, behavioral, and sexual health outcomes.

#### School drop-out

Four types of interventions (school support, economic strengthening, financial incentives, and asset building) with six studies were assessed for their impact on adolescents dropping out of school.^43^^,^^48^^,^^61^^,^^90^^,^^92^^,^^104^ Of those, school support (two studies; RR 0.36, 95% CI 0.26–0.50) and asset building (two studies; RR 0.33, 95% CI 0.28–0.39) significantly reduced the proportion of school drop-out rates among adolescents compared with a comparator (Table 6, Appendix p 26).

#### HIV stigma

A total of five studies were assessed for their impact on the total stigma (Four studies),^55^^,^^56^^,^^59^^,^^116^ self-stigma (four studies),^37^^,^^55^^,^^56^^,^^116^ and experienced stigma (three studies)^37^^,^^55^^,^^56^ among adolescents. Specifically, psychosocial support is associated with reduced reports of experienced stigma (one study; SMD −0.53, 95% CI −1.02 to −0.04) (Table 6, Appendix pp 27 and 28).

#### Transactional sex

Three types of interventions (school support, financial incentives, and asset building) with three studies were assessed for their impact on adolescents' transactional sex.^48^^,^^86^^,^^104^ Of those, school support was associated with the decreasing reports of transactional sex among adolescents compared with a comparator (one study; RR 0.53, 95% CI 0.32–0.88) (Table 6, Appendix p 29).

#### Condom use

Six types of interventions with nine studies were assessed for their impact on adolescents' condom usage.^42^^,^^48^^,^^56^^,^^80^^,^^86^^,^^88^^,^^89^^,^^104^^,^^114^ Among them, educational interventions (three studies; RR 1.08, 95% CI 1.02–1.16) and digital interventions (one study; RR 1.12, 95% CI 1.04–1.20) were associated with an increase in the proportion of condom usage among adolescents compared with a comparator (Table 6, Appendix p 30).

#### Adolescent pregnancy

Five types of interventions (school support, financial incentives, educational interventions, asset building, and m-Health) across seven studies were assessed for their effects on the prevalence of adolescent pregnancy.^48^^,^^61^^,^^90^^,^^104^^,^^113^^,^^114^^,^^126^ Among them, asset building (one study; RR 0.60, 95% CI 0.51–0.70) and school support (two studies; RR 0.52, 95% CI 0.31–0.88) significantly reduced the prevalence of pregnancy among adolescents compared with a comparator (Table 6, Appendix p 31).

#### STI Co-infection

Five types of interventions (school support, financial incentives, educational interventions, psychosocial support, and asset building) with eight studies were assessed for their impact on adolescents with both HIV and HSV-2 or other STI infections.^48^^,^^61^^,^^64^^,^^86^^,^^89^^,^^90^^,^^104^^,^^113^ Among them, only psychosocial support reported an association with lower STI co-infections among adolescents compared with a comparator (two studies; RR 0.33, 95% CI 0.13–0.89) (Table 6, Appendix p 32).

#### Sexual and reproductive health service utilization

Four types of interventions (multi-level intervention, mHealth, psychosocial support, and educational interventions) with seven studies were assessed for their impact on adolescents' utilization of sexual reproductive health (SRH) services.^35^^,^^36^^,^^52^^,^^67^^,^^114^^,^^123^^,^^126^ Among them, multi-level intervention (three studies; RR 3.73, 95% CI 1.65–8.45) and mHealth (two studies; RR 1.13, 95% CI 1.11–1.15) significantly increased the proportion of adolescents utilizing SRH services compared with a comparator (Table 6, Appendix p33).

Studies with different levels of adolescent engagement also experienced an influence on social outcomes. Adolescents with moderate/substantial levels of engagement experienced a significant decline in violence (two studies, RR 0.71, 95% CI 0.55–0.91) compared to those with minimum levels of engagement, which showed a non-significant result (two studies, RR 0.82, 95% CI 0.67–1.02). Additionally, higher levels of adolescent engagement corresponded to a significant increase in the proportion of adolescents utilizing sexual and reproductive health services (four studies, RR 2.29, 95% CI 1.52–3.45), while minimum engagement showed a relative risk that may not be significant (three studies, RR 1.14, 95% CI 0.96–1.34) (Appendix p16).

## Discussion

It is increasingly recognized that further progress in addressing the inequities and inequalities that persist across the entire HIV continuum will require keen attention to the social determinants of health.^130^ Intervention packages that allow the attainment of multiple outcomes across the care cascade offer the promise of efficiency in adolescent HIV programming. They should be a particularly appealing prospect for policymakers when they demonstrate multiple positive spillover effects; the mutualism and indivisibility of all outcomes is a fundamental principle inherent to the framing of the SDGs.

Our review found multi-outcome effects for a limited spectrum of interventions, notably differentiated service delivery models, asset-building, and digital interventions. Asset-building provides opportunities and fosters hope by enhancing individual, familial, and community resilience. Rather than direct financial assistance, asset-building involves cultivating emotional support, knowledge, and resources that empower individuals and communities to thrive. By combining asset-building with HIV prevention and care efforts, these interventions foster resilience and community cohesion while addressing the multifaceted challenges faced by vulnerable populations.^131^ Several studies in our review evaluated components of the DREAMS multi-country initiative, a complex multi-level, multi-layered asset-building intervention tailored for vulnerable adolescent girls.^132^ At the same time, similar approaches have been explored in other contexts. For example, community-based groups have been leveraged as platforms for HIV interventions, integrating HIV education, testing, and support services with financial literacy training and savings mobilization.^133^ Although asset-building has not been linked directly to decreases in HIV acquisition, interventions that build social capital have been shown to increase agency and improve HIV testing uptake among adolescent girls.^131^ Furthermore, asset-building interventions were associated with reductions in exposure to violence, dropping out from school, and the proportion of pregnancy among adolescents in this review.

In our review, most research interventions reported minimal to moderate adolescent engagement. This is consistent with data from two systematic reviews of community engagement in HIV trials, suggesting less robust youth engagement strategies.^4^^,^^134^ Many of the included studies had minimal youth engagement, especially in studies that incorporated clinical quality improvement, novel prevention options, and treatment as prevention. These findings highlight a significant gap in meaningful adolescent involvement in the design, implementation, and evaluation of HIV research interventions. Possible reasons for low engagement include ethical concerns about competing demands among youth, lack of youth training and capacity-building opportunities, adult perceptions about limited youth capacity, and a lack of parental consent due to the stigmatized nature of HIV.^4^ However, there were intervention areas that seemed to have registered success with youth engagement. In our systematic review, psychosocial, mental health, and digital research interventions were more likely to deploy moderate to substantive adolescent engagement. Another global crowdsourcing open call we conducted in parallel with this systematic review aimed to gather ideas from young people on how to improve HIV outcomes and broader adolescent well-being in high-HIV-burden countries, involving youth as co-researchers, and has also shown promise in fostering meaningful youth engagement.^135^ Our findings also indicate that increased and more active adolescent participation in multiple interventions may potentially lead to enhanced acceptability and effectiveness of these interventions. Increasing youth engagement in HIV intervention research is important for systematically ensuring the integration of the values, preferences, and choices favored by a diversity of adolescents in high-burden HIV countries.

This review has multiple implications for research and policy. From a research perspective, our findings highlight the urgency of prioritizing evidence generation on a wider array of interventions designed to serve adolescents with intentionality; there is a particular import to documenting areas where promising interventions can be effectively scaled for broader implementation. Additionally, there is a need for multi-dimensional approaches like asset-building, recognizing the interconnectedness of social determinants, broader well-being outcomes, and the HIV care cascade. There is a notable opportunity to document the specific system enhancements that enable health to be sustainably integrated and co-delivered with social care. One emerging area where we require more operations and intervention research is in the design and implementation of digitally-enabled interventions. Given the proliferation of digital health technologies and digitally enabled approaches, it is notable that we only found five studies evaluating the effects of digital interventions on adolescents’ HIV outcomes. It is important to note that certain interventions have been more frequently studied and shown to have benefits in adolescents, yet others are significantly under-represented in the literature. This publication bias does not imply that non-represented interventions are irrelevant to the population under consideration. Future research could explore the applicability of various interventions across different age groups, particularly for older adolescents who may be captured in studies alongside adults or within the broader youth category.

Our study suggests that robust adolescent-engaged research is feasible in a wide range of settings. Policymakers could consider integrating adolescent engagement strategies into comprehensive public health research tailored to adolescents in high-burden settings. Our findings highlight adolescent engagement as an important strategy to optimize implementation research. While we do not have evidence that programs lack substantive adolescent and youth engagement, our review identifies a gap in research engagement. This gap underscores the importance of focusing on strategies to better involve adolescents in research processes.

This study has several limitations. First, the comparator groups were often heterogeneous and poorly described, which could have impacted the efficacy of an intervention. High heterogeneity across interventions and outcomes may impact the generalizability of findings. Second, although we identified a greater number of randomized studies, a certain number of studies were still non-randomized, with little or no adjustment for confounding, reducing the data quality. Third, the risk of bias assessment revealed moderate to high concerns in some cases. While we did not exclude these studies to maintain a comprehensive review, we conducted sensitivity analyses to assess the impact of these biases on our overall findings. Fourth, adolescent engagement is difficult to measure, and some studies may have had more robust adolescent engagement than described. Additionally, the cyclical nature of the HIV cascade emphasizes that patient journeys are not strictly linear; individuals may move in and out of care at various points. This complexity suggests that interventions must not only focus on initial engagement but also prioritize strategies for re-engagement and continuity of care as patients navigate their health journey. Robust measures to better capture the dynamic nature of care engagement are wanting. This study did not capture data on individuals moving between these cascade steps, limiting our ability to fully understand these dynamics. Furthermore, our search and review yielded significantly more treatment studies and fewer studies on novel ARV-based prevention, which may be due to the exclusion of non-care cascade outcomes (e.g., risky sexual behavior and condom use). Lastly, although we screened 288 conference abstracts from Embase (2022–2023) to capture studies presented at conferences but not yet published, a broader search of grey literature could have identified additional relevant conference materials.

In conclusion, our study provides comprehensive insights into the landscape of HIV interventions for adolescents in high-burden countries. The diverse types of interventions assessed provide a foundation for future intervention development and research. Tailoring interventions to address specific priorities of adolescents and ensure their multi-dimensional well-being is important for advancing HIV prevention and care for this vulnerable population.

## Contributors

JT and DW conceptualized the study. JT, DW, YT, and MB designed the study. YT, LZ, ZZ, DH, OA, WS, UO, OA, MM, KA, JT, and IO reviewed and assessed the studies for inclusion. YT, DH, LZ, ZZ, BM, IA, OBA, OA, MM, and UO extracted data. BM, ZZ, DH, MM, and OA performed the risk of bias assessment. YT carried out analyses, and YT, MB, DW, and JT produced the first draft of the manuscript. All authors contributed to the interpretation of data and critical revisions of the manuscript. YT and DH have read and verified the underlying data within this manuscript. All authors have read and approved the final version of the manuscript. JT was responsible for the decision to submit the manuscript.

## Data sharing statement

The data analyzed during the current systematic review and meta-analysis are available from the corresponding author upon reasonable request.

## Declaration of interests

YT and MB received an honorarium for their work on this manuscript. DH has been paid through a K24 mentorship grant since 10/2023. All other authors declare no competing interests.
