# Supplementary material for: HIV interventions across the care continuum for adolescents in high-burden countries: a systematic review and meta-analysis
Source: eClinicalMedicine. 2024 Nov 28;78:102989. doi: 10.1016/j.eclinm.2024.102989 (PMC11701459; doi:10.1016/j.eclinm.2024.102989)
Supplement: Supplementary appendix [file mmc1.docx]

**HIV Interventions Across the Care Continuum for Adolescents in High-Burden Countries: A Systematic Review and Meta-Analysis**

**Supplementary appendix**

Table of contents

[Appendix Table 1. Search strategies used in each bibliographic database. 3](#_Toc180978289)

[Appendix Table 2. Glossary of intervention type definition. 8](#_Toc180978290)

[Appendix Table 3. Items used to assess the within-study bias of randomized studies of interventions. 10](#_Toc180978291)

[Appendix Table 4. Items used to assess the within-study bias of non-randomized studies of interventions. 12](#_Toc180978292)

[Appendix Table 5. Risk of bias assessments for randomized studies of interventions. 13](#_Toc180978293)

[Appendix Table 6. Risk of bias assessments for non-randomized studies of interventions. 15](#_Toc180978294)

[Appendix Table 7. Effect of different levels of adolescent engagement on the outcomes across the HIV prevention and care cascade and related social determinants outcomes. 16](#_Toc180978295)

[Appendix Figure 1. Forest plot examining the association between interventions and PrEP uptake. 17](#_Toc180978296)

[Appendix Figure 2. Funnel plot assessing publication bias for intervention studies on PrEP uptake 18](#_Toc180978297)

[Appendix Figure 3. Funnel plot assessing publication bias for intervention studies on HIV testing 19](#_Toc180978298)

[Appendix Figure 4. Funnel plot assessing publication bias for intervention studies on people’s awareness of HIV infections 20](#_Toc180978299)

[Appendix Figure 5. Funnel plot assessing publication bias for intervention studies on ART adherence 21](#_Toc180978300)

[Appendix Figure 6. Funnel plot assessing publication bias for intervention studies on retention in care 22](#_Toc180978301)

[Appendix Figure 7. Funnel plot assessing publication bias for intervention studies on virological suppression. 23](#_Toc180978302)

[Appendix Figure 9. Forest plot examining the association between interventions and violence experience. 25](#_Toc180978303)

[Appendix Figure 10. Forest plot examining the association between interventions and school drop-out. 26](#_Toc180978304)

[Appendix Figure 11. Forest plot examining the association between interventions and self-stigma. 27](#_Toc180978305)

[Appendix Figure 12. Forest plot examining the association between interventions and experienced stigma. 28](#_Toc180978306)

[Appendix Figure 13. Forest plot examining the association between interventions and transactional sex. 29](#_Toc180978307)

[Appendix Figure 14. Forest plot examining the association between interventions and condom usage. 30](#_Toc180978308)

[Appendix Figure 15. Forest plot examining the association between interventions and adolescent pregnancy. 31](#_Toc180978309)

[Appendix Figure 16. Forest plot examining the association between interventions and STI Co-infection. 32](#_Toc180978310)

[Appendix Figure 17. Forest plot examining the association between interventions and sexual and reproductive health service utilization. 33](#_Toc180978311)

# **Appendix Table 1.** Search strategies used in each bibliographic database.

**Database: PubMed**

| **Set #** |  | **Results** |
| --- | --- | --- |
| 1 | "Angola"[Mesh] OR "Bangladesh"[Mesh] OR "Botswana"[Mesh] OR "Brazil"[Mesh] OR "Burundi"[Mesh] OR "Cameroon"[Mesh] OR "Chad"[Mesh] OR "China"[Mesh] OR "Democratic Republic of the Congo"[Mesh] OR "Cote d'Ivoire"[Mesh] OR "Djibouti"[Mesh] OR "Dominican Republic"[Mesh] OR "Eswatini"[Mesh] OR "Ethiopia"[Mesh] OR "Ghana"[Mesh] OR "Haiti"[Mesh] OR "India"[Mesh] OR "Indonesia"[Mesh] OR "Iran"[Mesh] OR "Kenya"[Mesh] OR "Lesotho"[Mesh] OR "Malawi"[Mesh] OR "Mozambique"[Mesh] OR "Myanmar"[Mesh] OR "Namibia"[Mesh] OR "Nigeria"[Mesh] OR "Pakistan"[Mesh] OR "Papua New Guinea"[Mesh] OR "Philippines"[Mesh] OR "Rwanda"[Mesh] OR "South Africa"[Mesh] OR "Tanzania"[Mesh] OR "Uganda"[Mesh] OR "Ukraine"[Mesh] OR "Uzbekistan"[Mesh] OR "Zambia"[Mesh] OR "Zimbabwe"[Mesh] OR Angola[tiab] OR Angolan[tiab] OR Angolans[tiab] OR Bangladesh[tiab] OR Bangladeshi[tiab] OR Bangladeshis[tiab] OR Botswana[tiab] OR Motswana[tiab] OR Batswana[tiab] OR Brazil[tiab] OR Brazilian[tiab] OR Brazilians[tiab] OR Burundi[tiab] OR Burundian[tiab] OR Burundians[tiab] OR Cameroon[tiab] OR Cameroonian[tiab] OR Cameroonians[tiab] OR Chad[tiab] OR Chadian[tiab] OR Chadians[tiab] OR China[tiab] OR Chinese[tiab] OR “Côte d'Ivoire” [tiab] OR Ivorian[tiab] OR Ivorians[tiab] OR "Democratic Republic of the Congo"[tiab] OR Congolese[tiab] OR Djibouti[tiab] OR Djiboutian[tiab] OR Djiboutians[tiab] OR “Dominican Republic” [tiab] OR Dominican[tiab] OR Dominicans[tiab] OR Eswatini[tiab] OR Swazi[tiab] OR Emaswati[tiab] OR Liswati[tiab] OR Ethiopia[tiab] OR Ethiopian[tiab] OR Ethiopians[tiab] OR Ghana[tiab] OR Ghanaian[tiab] OR Ghanaians[tiab] OR Haiti[tiab] OR Haitian[tiab] OR Haitians[tiab] OR India[tiab] OR Indian[tiab] OR Indians[tiab] OR Indonesia[tiab] OR Indonesian[tiab] OR Indonesians[tiab] OR Iran[tiab] OR Iranian[tiab] OR Iranians[tiab] OR Kenya[tiab] OR Kenyan[tiab] OR Kenyans[tiab] OR Lesotho[tiab] OR Mosotho[tiab] OR Basotho[tiab] OR Malawi[tiab] OR Malawian[tiab] OR Malawians[tiab] OR Mozambique[tiab] OR Mozambican[tiab] OR Mozambicans[tiab] OR Myanmar[tiab] OR Burmese[tiab] OR Myanma[tiab] OR Namibia[tiab] OR Namibian[tiab] OR Namibians[tiab] OR Nigeria[tiab] OR Nigerian[tiab] OR Nigerians[tiab] OR Pakistan[tiab] OR Pakistani[tiab] OR Pakistanis[tiab] OR “Papua New Guinea”[tiab] OR “Papua New Guinean”[tiab] OR “Papua New Guineans”[tiab] OR Philippines[tiab] OR Filipino[tiab] OR Filipinos[tiab] OR Filipina[tiab] OR Filipinas[tiab] OR Philippine[tiab] OR Rwanda[tiab] OR Rwandan[tiab] OR Rwandese[tiab] OR “South Africa”[tiab] OR “South African”[tiab] OR “South Africans”[tiab] OR Tanzania[tiab] OR Tanzanian[tiab] OR Tanzanians[tiab] OR Uganda[tiab] OR Ugandan[tiab] OR Ugandans[tiab] OR Ukraine[tiab] OR Ukrainian[tiab] OR Ukrainians[tiab] OR Uzbekistan[tiab] OR Uzbekistani[tiab] OR Uzbekistanis[tiab] OR Zambia[tiab] OR Zambian[tiab] OR Zambians[tiab] OR Zimbabwe[tiab] OR Zimbabwean[tiab] OR Zimbabweans[tiab] OR Zimbo[tiab] |  |
| 2 | "Adolescent"[Mesh] OR adolescent[tiab] OR adolescents[tiab] OR adolescence[tiab] OR youth[tiab] OR youths[tiab] OR teenager[tiab] OR teenagers[tiab] OR teenaged[tiab] OR teenage[tiab] OR teen[tiab] OR teens[tiab] OR girl[tiab] OR girls[tiab] OR boy[tiab] OR boys[tiab] OR preadolescent[tiab] OR preadolescents[tiab] OR prepubescent[tiab] OR AYA[tiab] OR "Young Adult"[Mesh] OR “young adult”[tiab] OR “young adults”[tiab] OR “young adulthood”[tiab] |  |
| 3 | "HIV infections"[mesh] OR HIV[mesh] OR "Acquired Immunodeficiency Syndrome"[mesh] OR HIV[tiab] OR "human immunodeficiency virus"[tiab] OR AIDS[tiab] OR "Acquired Immunodeficiency Syndrome"[tiab] |  |
| 4 | “randomized controlled trial”[pt] OR “controlled clinical trial”[pt] OR randomized[tiab] OR randomised[tiab] OR randomization[tiab] OR randomisation[tiab] OR randomly[tiab] OR trial[tiab] OR groups[tiab] OR "Comparative Study"[Publication Type] OR "Controlled Clinical Trial"[Publication Type] OR Nonrandom[tiab] OR non-random[tiab] OR nonrandomized[tiab] OR non-randomized[tiab] OR nonrandomized[tiab] OR non-randomised[tiab] OR quasi-experiment*[tiab] OR quasiexperiment*[tiab] OR quasirandom*[tiab] OR quasi-random*[tiab] OR quasi-control*[tiab] OR quasicontrol*[tiab] NOT (animals[mh] NOT humans[mh]) NOT (Editorial[ptyp] OR Letter[ptyp] OR Case Reports[ptyp] OR Comment[ptyp]) |  |
| 5 | #1 AND #2 AND #3 AND #4 | 7322 |
| 6 | #5 AND English[lang] | 7121 |
| 7 | #6 AND ("2015"[Date - Publication]: "3000"[Date - Publication]) | 3346 |

**Database: Scopus**

| Set # |  | Results |
| --- | --- | --- |
| 1 | TITLE-ABS (angola OR angolan OR angolans OR bangladesh OR bangladeshi OR bangladeshis OR botswana OR motswana OR batswana OR brazil OR brazilian OR brazilians OR burundi OR burundian OR burundians OR cameroon OR cameroonian OR cameroonians OR chad OR chadian OR chadians OR china OR chinese OR "Côte d&apos;Ivoire" OR ivorian OR ivorians OR "Democratic Republic of the Congo" OR congolese OR djibouti OR djiboutian OR djiboutians OR "Dominican Republic" OR dominican OR dominicans OR eswatini OR swazi OR emaswati OR liswati OR ethiopia OR ethiopian OR ethiopians OR ghana OR ghanaian OR ghanaians OR haiti OR haitian OR haitians OR india OR indian OR indians OR indonesia OR indonesian OR indonesians OR iran OR iranian OR iranians OR kenya OR kenyan OR kenyans OR lesotho OR mosotho OR basotho OR malawi OR malawian OR malawians OR mozambique OR mozambican OR mozambicans OR myanmar OR burmese OR myanma OR namibia OR namibian OR namibians OR nigeria OR nigerian OR nigerians OR pakistan OR pakistani OR pakistanis OR "Papua New Guinea" OR "Papua New Guinean" OR "Papua New Guineans" OR philippines OR filipino OR filipinos OR filipina OR filipinas OR philippine OR rwanda OR rwandan OR rwandese OR "South Africa" OR "South African" OR "South Africans" OR tanzania OR tanzanian OR tanzanians OR uganda OR ugandan OR ugandans OR ukraine OR ukrainian OR ukrainians OR uzbekistan OR uzbekistani OR uzbekistanis OR zambia OR zambian OR zambians OR zimbabwe OR zimbabwean OR zimbabweans OR zimbo) |  |
| 2 | TITLE-ABS ( adolescent OR adolescents OR adolescence OR youth OR youths OR teenager OR teenagers OR teenaged OR teenage OR teen OR teens OR girl OR girls OR boy OR boys OR preadolescent OR preadolescents OR prepubescent OR aya OR "young adult" OR "young adults" OR "young adulthood") |  |
| 3 | TITLE-ABS (hiv OR "human immunodeficiency virus" OR aids OR "Acquired Immunodeficiency Syndrome") |  |
| 4 | TITLE-ABS (randomized OR randomized OR randomization OR randomization OR randomly OR trial OR groups OR Nonrandom OR non-random OR non-randomized OR non-randomized OR non-randomized OR non-randomized OR quasi-experiment* OR quasiexperiment* OR quasirandom* OR quasi-random* OR quasi-control* OR quasicontrol*) |  |
| 5 | #1 AND #2 AND #3 AND #4 | 2811 |
| 6 | #5 AND ( LIMIT-TO ( LANGUAGE, "English")) | 2750 |
| 7 | #6 AND ( LIMIT-TO (PUBYEAR, 2023) OR LIMIT-TO (PUBYEAR, 2022) OR LIMIT-TO (PUBYEAR, 2021) OR LIMIT-TO (PUBYEAR, 2020) OR LIMIT-TO (PUBYEAR, 2019) OR LIMIT-TO (PUBYEAR, 2018) OR LIMIT-TO (PUBYEAR, 2017) OR LIMIT-TO (PUBYEAR, 2016) OR LIMIT-TO (PUBYEAR, 2015)) | 1528 |

**Database: Embase (Elsevier)**

| Set # |  | Results |
| --- | --- | --- |
| 1 | 'Angola'/exp OR 'Bangladesh'/exp OR 'Botswana'/exp OR 'Brazil'/exp OR 'Burundi'/exp OR 'Cameroon'/exp OR 'Chad'/exp OR 'China'/exp OR 'Democratic Republic Congo'/exp OR 'Cote d`Ivoire'/exp OR 'Djibouti'/exp OR 'Dominican Republic'/exp OR 'Eswatini'/exp OR 'Ethiopia'/exp OR 'Ghana'/exp OR 'Haiti'/exp OR 'India'/exp OR 'Indonesia'/exp OR 'Iran'/exp OR 'Kenya'/exp OR 'Lesotho'/exp OR 'Malawi'/exp OR 'Mozambique'/exp OR 'Myanmar'/exp OR 'Namibia'/exp OR 'Nigeria'/exp OR 'Pakistan'/exp OR 'Papua New Guinea'/exp OR 'Philippines'/exp OR 'Rwanda'/exp OR 'South Africa'/exp OR 'Tanzania'/exp OR 'Uganda'/exp OR 'Ukraine'/exp OR 'Uzbekistan'/exp OR 'Zambia'/exp OR 'Zimbabwe'/exp OR Angola:ti,ab,kw OR Angolan:ti,ab,kw OR Angolans:ti,ab,kw OR Bangladesh:ti,ab,kw OR Bangladeshi:ti,ab,kw OR Bangladeshis:ti,ab,kw OR Botswana:ti,ab,kw OR Motswana:ti,ab,kw OR Batswana:ti,ab,kw OR Brazil:ti,ab,kw OR Brazilian:ti,ab,kw OR Brazilians:ti,ab,kw OR Burundi:ti,ab,kw OR Burundian:ti,ab,kw OR Burundians:ti,ab,kw OR Cameroon:ti,ab,kw OR Cameroonian:ti,ab,kw OR Cameroonians:ti,ab,kw OR Chad:ti,ab,kw OR Chadian:ti,ab,kw OR Chadians:ti,ab,kw OR China:ti,ab,kw OR Chinese:ti,ab,kw OR ‘Cote dIvoire’:ti,ab,kw OR Ivorian:ti,ab,kw OR Ivorians:ti,ab,kw OR ‘Democratic Republic of the Congo’:ti,ab,kw OR Congolese:ti,ab,kw OR Djibouti:ti,ab,kw OR Djiboutian:ti,ab,kw OR Djiboutians:ti,ab,kw OR ‘Dominican Republic’:ti,ab,kw OR Dominican:ti,ab,kw OR Dominicans:ti,ab,kw OR Eswatini:ti,ab,kw OR Swazi:ti,ab,kw OR Emaswati:ti,ab,kw OR Liswati:ti,ab,kw OR Ethiopia:ti,ab,kw OR Ethiopian:ti,ab,kw OR Ethiopians:ti,ab,kw OR Ghana:ti,ab,kw OR Ghanaian:ti,ab,kw OR Ghanaians:ti,ab,kw OR Haiti:ti,ab,kw OR Haitian:ti,ab,kw OR Haitians:ti,ab,kw OR India:ti,ab,kw OR Indian:ti,ab,kw OR Indians:ti,ab,kw OR Indonesia:ti,ab,kw OR Indonesian:ti,ab,kw OR Indonesians:ti,ab,kw OR Iran:ti,ab,kw OR Iranian:ti,ab,kw OR Iranians:ti,ab,kw OR Kenya:ti,ab,kw OR Kenyan:ti,ab,kw OR Kenyans:ti,ab,kw OR Lesotho:ti,ab,kw OR Mosotho:ti,ab,kw OR Basotho:ti,ab,kw OR Malawi:ti,ab,kw OR Malawian:ti,ab,kw OR Malawians:ti,ab,kw OR Mozambique:ti,ab,kw OR Mozambican:ti,ab,kw OR Mozambicans:ti,ab,kw OR Myanmar:ti,ab,kw OR Burmese:ti,ab,kw OR Myanma:ti,ab,kw OR Namibia:ti,ab,kw OR Namibian:ti,ab,kw OR Namibians:ti,ab,kw OR Nigeria:ti,ab,kw OR Nigerian:ti,ab,kw OR Nigerians:ti,ab,kw OR Pakistan:ti,ab,kw OR Pakistani:ti,ab,kw OR Pakistanis:ti,ab,kw OR ‘Papua New Guinea’:ti,ab,kw OR ‘Papua New Guinean’:ti,ab,kw OR ‘Papua New Guineans’:ti,ab,kw OR Philippines:ti,ab,kw OR Filipino:ti,ab,kw OR Filipinos:ti,ab,kw OR Filipina:ti,ab,kw OR Filipinas:ti,ab,kw OR Philippine:ti,ab,kw OR Rwanda:ti,ab,kw OR Rwandan:ti,ab,kw OR Rwandese:ti,ab,kw OR ‘South Africa’:ti,ab,kw OR ‘South African’:ti,ab,kw OR ‘South Africans’:ti,ab,kw OR Tanzania:ti,ab,kw OR Tanzanian:ti,ab,kw OR Tanzanians:ti,ab,kw OR Uganda:ti,ab,kw OR Ugandan:ti,ab,kw OR Ugandans:ti,ab,kw OR Ukraine:ti,ab,kw OR Ukrainian:ti,ab,kw OR Ukrainians:ti,ab,kw OR Uzbekistan:ti,ab,kw OR Uzbekistani:ti,ab,kw OR Uzbekistanis:ti,ab,kw OR Zambia:ti,ab,kw OR Zambian:ti,ab,kw OR Zambians:ti,ab,kw OR Zimbabwe:ti,ab,kw OR Zimbabwean:ti,ab,kw OR Zimbabweans:ti,ab,kw OR Zimbo:ti,ab,kw |  |
| 2 | 'adolescent'/exp OR 'young adult'/exp OR adolescent:ti,ab,kw OR adolescents:ti,ab,kw OR adolescence:ti,ab,kw OR youth:ti,ab,kw OR youths:ti,ab,kw OR teenager:ti,ab,kw OR teenagers:ti,ab,kw OR teenaged:ti,ab,kw OR teenage:ti,ab,kw OR teen:ti,ab,kw OR teens:ti,ab,kw OR girl:ti,ab,kw OR girls:ti,ab,kw OR boy:ti,ab,kw OR boys:ti,ab,kw OR preadolescent:ti,ab,kw OR preadolescents:ti,ab,kw OR prepubescent:ti,ab,kw OR AYA:ti,ab,kw OR ‘young adult’:ti,ab,kw OR ‘young adults’:ti,ab,kw OR ‘young adulthood’:ti,ab,kw |  |
| 3 | 'Human immunodeficiency virus infection'/exp OR 'Human immunodeficiency virus'/exp OR 'acquired immune deficiency syndrome'/exp OR HIV:ti,ab,kw OR ‘human immunodeficiency virus’:ti,ab,kw OR AIDS:ti,ab,kw OR ‘Acquired Immunodeficiency Syndrome’:ti,ab,kw |  |
| 4 | 'randomized controlled trial'/exp OR 'crossover procedure'/exp OR 'double blind procedure'/exp OR 'single blind procedure'/exp OR random* OR factorial* OR crossover* OR cross NEAR/1 over* OR placebo* OR doubl* NEAR/1 blind* OR singl* NEAR/1 blind* OR assign* OR allocat* OR volunteer* OR 'clinical study'/exp OR ‘clinical trial’:ti,ab OR ‘clinical trials’:ti,ab OR 'controlled study'/exp OR 'comparative study'/exp OR Nonrandom:ti,ab,kw OR non-random:ti,ab,kw OR non-randomized:ti,ab,kw OR non-randomized:ti,ab,kw OR non-randomized:ti,ab,kw OR non-randomized:ti,ab,kw OR quasi-experiment*:ti,ab,kw OR quasiexperiment*:ti,ab,kw OR quasirandom*:ti,ab,kw OR quasi-random*:ti,ab,kw OR quasi-control*:ti,ab,kw OR quasicontrol*:ti,ab,kw |  |
| 5 | #1 AND #2 AND #3 AND #4 | 21346 |
| 6 | #5 AND [english]/lim | 20880 |
| 7 | #6 AND [2015-2023]/py | 12468 |
| 8 | #7 AND [embase]/lim NOT ([embase]/lim AND [medline]/lim) | 4500 |

**Database: Cochrane Library**

| Set # |  | Results |
| --- | --- | --- |
| 1 | [mh "Angola"] OR [mh "Bangladesh"] OR [mh "Botswana"] OR [mh "Brazil"] OR [mh "Burundi"] OR [mh "Cameroon"] OR [mh "Chad"] OR [mh "China"] OR [mh "Democratic Republic of the Congo"] OR [mh "Cote d'Ivoire"] OR [mh "Djibouti"] OR [mh "Dominican Republic"] OR [mh "Eswatini"] OR [mh "Ethiopia"] OR [mh "Ghana"] OR [mh "Haiti"] OR [mh "India"] OR [mh "Indonesia"] OR [mh "Iran"] OR [mh "Kenya"] OR [mh "Lesotho"] OR [mh "Malawi"] OR [mh "Mozambique"] OR [mh "Myanmar"] OR [mh "Namibia"] OR [mh "Nigeria"] OR [mh "Pakistan"] OR [mh "Papua New Guinea"] OR [mh "Philippines"] OR [mh "Rwanda"] OR [mh "South Africa"] OR [mh "Tanzania"] OR [mh "Uganda"] OR [mh "Ukraine"] OR [mh "Uzbekistan"] OR [mh "Zambia"] OR [mh "Zimbabwe"] OR Angola:ti,ab OR Angolan:ti,ab OR Angolans:ti,ab OR Bangladesh:ti,ab OR Bangladeshi:ti,ab OR Bangladeshis:ti,ab OR Botswana:ti,ab OR Motswana:ti,ab OR Batswana:ti,ab OR Brazil:ti,ab OR Brazilian:ti,ab OR Brazilians:ti,ab OR Burundi:ti,ab OR Burundian:ti,ab OR Burundians:ti,ab OR Cameroon:ti,ab OR Cameroonian:ti,ab OR Cameroonians:ti,ab OR Chad:ti,ab OR Chadian:ti,ab OR Chadians:ti,ab OR China:ti,ab OR Chinese:ti,ab OR “Côte d'Ivoire”:ti,ab OR Ivorian:ti,ab OR Ivorians:ti,ab OR "Democratic Republic of the Congo":ti,ab OR Congolese:ti,ab OR Djibouti:ti,ab OR Djiboutian:ti,ab OR Djiboutians:ti,ab OR “Dominican Republic”:ti,ab OR Dominican:ti,ab OR Dominicans:ti,ab OR Eswatini:ti,ab OR Swazi:ti,ab OR Emaswati:ti,ab OR Liswati:ti,ab OR Ethiopia:ti,ab OR Ethiopian:ti,ab OR Ethiopians:ti,ab OR Ghana:ti,ab OR Ghanaian:ti,ab OR Ghanaians:ti,ab OR Haiti:ti,ab OR Haitian:ti,ab OR Haitians:ti,ab OR India:ti,ab OR Indian:ti,ab OR Indians:ti,ab OR Indonesia:ti,ab OR Indonesian:ti,ab OR Indonesians:ti,ab OR Iran:ti,ab OR Iranian:ti,ab OR Iranians:ti,ab OR Kenya:ti,ab OR Kenyan:ti,ab OR Kenyans:ti,ab OR Lesotho:ti,ab OR Mosotho:ti,ab OR Basotho:ti,ab OR Malawi:ti,ab OR Malawian:ti,ab OR Malawians:ti,ab OR Mozambique:ti,ab OR Mozambican:ti,ab OR Mozambicans:ti,ab OR Myanmar:ti,ab OR Burmese:ti,ab OR Myanma:ti,ab OR Namibia:ti,ab OR Namibian:ti,ab OR Namibians:ti,ab OR Nigeria:ti,ab OR Nigerian:ti,ab OR Nigerians:ti,ab OR Pakistan:ti,ab OR Pakistani:ti,ab OR Pakistanis:ti,ab OR “Papua New Guinea”:ti,ab OR “Papua New Guinean”:ti,ab OR “Papua New Guineans”:ti,ab OR Philippines:ti,ab OR Filipino:ti,ab OR Filipinos:ti,ab OR Filipina:ti,ab OR Filipinas:ti,ab OR Philippine:ti,ab OR Rwanda:ti,ab OR Rwandan:ti,ab OR Rwandese:ti,ab OR “South Africa”:ti,ab OR “South African”:ti,ab OR “South Africans”:ti,ab OR Tanzania:ti,ab OR Tanzanian:ti,ab OR Tanzanians:ti,ab OR Uganda:ti,ab OR Ugandan:ti,ab OR Ugandans:ti,ab OR Ukraine:ti,ab OR Ukrainian:ti,ab OR Ukrainians:ti,ab OR Uzbekistan:ti,ab OR Uzbekistani:ti,ab OR Uzbekistanis:ti,ab OR Zambia:ti,ab OR Zambian:ti,ab OR Zambians:ti,ab OR Zimbabwe:ti,ab OR Zimbabwean:ti,ab OR Zimbabweans:ti,ab OR Zimbo:ti,ab |  |
| 2 | [mh "Adolescent"] OR [mh "Young Adult"] OR adolescent:ti,ab OR adolescents:ti,ab OR adolescence:ti,ab OR youth:ti,ab OR youths:ti,ab OR teenager:ti,ab OR teenagers:ti,ab OR teenaged:ti,ab OR teenage:ti,ab OR teen:ti,ab OR teens:ti,ab OR girl:ti,ab OR girls:ti,ab OR boy:ti,ab OR boys:ti,ab OR preadolescent:ti,ab OR preadolescents:ti,ab OR prepubescent:ti,ab OR AYA:ti,ab OR “young adult”:ti,ab OR “young adults”:ti,ab OR “young adulthood”:ti,ab |  |
| 3 | [mh "HIV infections"] OR [mh HIV] OR [mh "Acquired Immunodeficiency Syndrome"] OR HIV:ti,ab OR "human immunodeficiency virus":ti,ab OR AIDS:ti,ab OR "Acquired Immunodeficiency Syndrome":ti,ab |  |
| 4 | #1 AND #2 AND #3 |  |
| 5 | #4 AND published since 2015 | 1337 |

# **Appendix Table 2.** Glossary of intervention type definition.

| **Intervention type** | **Definition** |
| --- | --- |
| School support | Financial assistance programs aimed at reducing school drop-out rates among adolescents affected by HIV. These programs may provide subsidies for school fees, uniforms, textbooks, and other educational expenses to alleviate financial barriers to education. |
| Educational interventions | Programs focused on enhancing knowledge, skills, and awareness about HIV, sexual health, and related topics to empower individuals, particularly adolescents, to make informed health decisions. These interventions often include workshops, group discussions, and multimedia resources and may be delivered in schools, community centers, or health facilities. |
| Point-of-care viral load and drug resistance mutation testing | On-site testing methods allow for rapid viral load and drug resistance assessment at the location of care. This approach reduces the time needed to obtain results, enabling more timely adjustments to treatment regimens and improving viral suppression rates by identifying resistance patterns early. |
| Novel prevention options | Innovative strategies and tools aimed at reducing HIV transmission risk, including options like pre-exposure prophylaxis (PrEP), long-acting injectable treatments, microbicides, and new vaccine developments. These prevention methods are tailored to provide diverse, accessible choices for different populations at risk. |
| Prevention of mother-to-child transmission | Comprehensive interventions to prevent HIV transmission from HIV-positive mothers to their infants during pregnancy, childbirth, or breastfeeding. These programs include antiretroviral therapy for mothers, safe delivery practices, and infant feeding guidelines, aiming to reduce the risk of vertical transmission to near zero. |
| Targeted contact-based HIV testing interventions | Testing strategies focus on identifying and testing close contacts of individuals diagnosed with HIV, such as family members, partners, and other high-risk groups. This targeted approach aims to improve early HIV detection and linkage to care among those most at risk. |
| Differentiated service delivery | Tailoring HIV-related healthcare services to meet the diverse needs and preferences of individuals or communities affected by HIV. This approach involves providing services adapted based on factors such as HIV status, stage of infection, treatment regimen, and social context to improve access, adherence, and outcomes. |
| Multi-level interventions | Strategies that address HIV prevention, treatment, and care across multiple levels of influence, including individual, interpersonal, community, and structural factors. These interventions recognize that effective HIV response requires action at various levels to create supportive environments, change behaviors, and reduce transmission. |
| Mental health interventions | Interventions designed to address the psychological, emotional, and social well-being of individuals living with HIV or affected by HIV include counseling, therapy, support groups, and psychosocial support services. These interventions aim to alleviate stress, depression, anxiety, and other mental health issues associated with HIV. |
| Treatment as prevention | Treatment as Prevention (TasP) refers to taking HIV medicine to prevent the sexual transmission of HIV. It is one of the most highly effective options for preventing HIV transmission. |
| Asset building | Enhancing the social, financial, and educational resources available to adolescents, such as skills training programs, financial literacy education, initiatives to improve their access to education and employment opportunities, and related. |
| Self-care interventions | Strategies that empower individuals living with HIV to take an active role in managing their own health and well-being, including self-testing, medication adherence, symptom management, healthy lifestyle choices, and self-monitoring practices |
| Financial incentives | Providing monetary rewards or benefits to encourage HIV prevention behaviors (e.g., condom use, HIV testing), linkage to care, treatment adherence, viral suppression, or participation in research studies or programs aimed at reducing HIV transmission and improving health outcomes. |
| Economic strengthening | Programs and initiatives aimed at improving the economic well-being and livelihoods of people living with HIV or affected by HIV. This may involve income-generating activities, vocational training, microfinance, job placement services, and financial literacy training to enhance financial stability and resilience. |
| Psychosocial support | Interventions that address the psychological and social aspects of living with HIV, including stigma reduction, disclosure support, coping skills training, peer support programs, and community-based interventions to enhance social support networks and resilience. |
| Digital Interventions | Using online platforms, telehealth, telemedicine, wearables, connectivity, mobile applications, personalized care, and other internet-enabled tools to provide adolescents with health information, support services, feedback elicitation, and engagement. |
| Mobile phone (calls or SMS) | Using mobile devices such as smartphones and tablets to deliver health-related messages, reminders, and support for treatment adherence through SMS, apps, and other mobile technologies. |
| Disclosure support | Providing guidance, counseling, and support to individuals living with HIV who are considering or navigating the process of disclosing their HIV status to partners, family members, friends, or healthcare providers to promote informed decision-making, reduce stigma, and enhance social support. |
| Clinical quality improvement | Initiatives aimed at enhancing the quality, efficiency, and effectiveness of HIV-related services delivered within healthcare settings, including clinics, hospitals, community health centers, and HIV treatment facilities, to ensure optimal clinical outcomes and patient satisfaction. |

# **Appendix Table 3.** Items used to assess the within-study bias of randomized studies of interventions.

| **Bias domain and signaling question*** | **Response options** | | |
| --- | --- | --- | --- |
|  | **Lower risk of bias** | **Higher risk of bias** | **Other** |
| **1. Bias arising from the randomization process** |  |  |  |
| 1.1 Was the allocation sequence random? | Y/PY | N/PN | NI |
| 1.2 Was the allocation sequence concealed until participants were enrolled and assigned to interventions? | Y/PY | N/PN | NI |
| 1.3 Did baseline differences between intervention groups suggest a problem with the randomization process? | N/PN | Y/PY | NI |
| Risk-of-bias judgment (low/high/some concerns) |  |  |  |
| Optional: What is the predicted direction of bias arising from the randomization process? |  |  |  |
| **2. Bias due to deviations from intended interventions** |  |  |  |
| 2.1 Were participants aware of their assigned intervention during the trial? | N/PN | Y/PY | NI |
| 2.2 Were carers and people delivering the interventions aware of participants’ assigned intervention during the trial? | N/PN | Y/PY | NI |
| 2.3 If Y/PY/NI to 2.1 or 2.2: Were there deviations from the intended intervention that arose because of the trial context? | N/PN | Y/PY | NA/NI |
| 2.4 If Y/PY/NI to 2.3: Were these deviations likely to have affected the outcome? | N/PN | Y/PY | NA/NI |
| 2.5 If Y/PY to 2.4: Were these deviations from the intended intervention balanced between groups? | Y/PY | N/PN | NA/NI |
| 2.6 Was an appropriate analysis used to estimate the effect of assignment to intervention? | Y/PY | N/PN | NI |
| 2.7 If N/PN/NI to 2.6: Was there potential for a substantial impact (on the result) of the failure to analyze participants in the group to which they were randomized? | N/PN | Y/PY | NA/NI |
| Risk-of-bias judgment (low/high/some concerns) |  |  |  |
| Optional: What is the predicted direction of bias due to deviations from intended interventions? |  |  |  |
| **3. Bias due to missing outcome data** |  |  |  |
| 3.1 Were data for this outcome available for all, or nearly all, participants randomized? | Y/PY | N/PN | NI |
| 3.2 If N/PN/NI to 3.1: Is there evidence that the result was not biased by missing outcome data? | Y/PY | N/PN | NA |
| 3.3 If N/PN to 3.2: Could missingness in the outcome depend on its true value? | N/PN | Y/PY | NA/NI |
| 3.4 If Y/PY/NI to 3.3: Is it likely that missingness in the outcome depended on its true value? | N/PN | Y/PY | NA/NI |
| Risk-of-bias judgment (low/high/some concerns) |  |  |  |
| Optional: What is the predicted direction of bias due to missing outcome data? |  |  |  |
| **4. Bias in the measurement of the outcome** |  |  |  |
| 4.1 Was the method of measuring the outcome inappropriate? | N/PN | Y/PY | NI |
| 4.2 Could measurement or ascertainment of the outcome have differed between intervention groups? | N/PN | Y/PY | NI |
| 4.3 If N/PN/NI to 4.1 and 4.2: Were outcome assessors aware of the intervention received by study participants? | N/PN | Y/PY | NI |
| 4.4 If Y/PY/NI to 4.3: Could assessment of the outcome have been influenced by knowledge of intervention received? | N/PN | Y/PY | NA/NI |
| 4.5 If Y/PY/NI to 4.4: Is it likely that assessment of the outcome was influenced by knowledge of intervention received? | N/PN | Y/PY | NA/NI |
| Risk-of-bias judgment (low/high/some concerns) |  |  |  |
| Optional: What is the predicted direction of bias in the measurement of the outcome? |  |  |  |
| **5. Bias in the selection of the reported result** |  |  |  |
| 5.1 Were the data that produced this result analyzed in accordance with a pre-specified analysis plan that was finalized before unblinded outcome data were available for analysis? | Y/PY | N/PN | NI |
| Is the numerical result being assessed likely to have been selected, on the basis of the results, from: |  |  |  |
| 5.2 ... multiple eligible outcome measurements (e.g., scales, definitions, time points) within the outcome domain? | N/PN | Y/PY | NI |
| 5.3 ... multiple eligible analyses of the data? | N/PN | Y/PY | NI |
| Risk-of-bias judgment (low/high/some concerns) |  |  |  |
| Optional: What is the predicted direction bias due to the selection of the reported results? |  |  |  |
| **Overall bias** |  |  |  |
| Risk-of-bias judgment (low/high/some concerns) |  |  |  |
| Optional: What is the overall predicted direction of bias for this outcome? |  |  |  |

Y=yes; PY=probably yes; PN=probably no; N=no; NA=not applicable; NI=no information.

*Signalling questions for bias due to deviations from intended interventions relate to the effect of assignment to intervention

| **Overall risk-of-bias Judgment Criteria** |
| --- |
| **Low risk of bias:** The study is judged to be at low risk of bias for all domains for this result |
| **Some concerns:** The study is judged to raise some concerns in at least one domain for this result but not to be at high risk of bias for any domain |
| **High risk of bias:** The study is judged to be at high risk of bias in at least one domain for this result, or the study is judged to have some concerns for multiple domains in a way that substantially lowers confidence in the result |

# **Appendix Table 4.** Items used to assess the within-study bias of non-randomized studies of interventions.

| **Criteria** |
| --- |
| **1. Selection bias** |
| - 1. Was the study’s target population pre-specified and appropriately chosen to answer the study question(s)?   1.2. Was the sampling frame a true or close representation of the target population? |
| 1.3. Was prospective selection used to select the sample? |
| **2. Bias due to missing outcome data** |
| 2.1 Was the proportion of missing outcome data minimal, and were there no/minimal differences between those with and without missing outcome data? |
| **3. Bias in the measurement of the outcome** |
| 3.1. Were data collected directly from the subjects (as opposed to a proxy?) |
| 3.2. Was an acceptable case definition used in the study? |
| 3.3. Was the study instrument that measured the parameter of interest (e.g., the proportion of results obtained on the same day) shown to have reliability and validity? |
| 3.4. Was the same mode of data collection used for all subjects? |
| 3.5. Was the length of the shortest measurement period appropriate for the parameter of interest? |
| **4. Bias in analysis** |
| 4.1. Were the numerator(s) and denominator(s) for the parameter of interest appropriate? |

Y=yes; N=no; NA=not applicable; NI=no information.

| **Overall judgment of risk of bias:** |
| --- |
| Low risk: all domains indicated as low risk |
| Some/medium risk: 1 or no domains have signaling questions indicating high risk; OR, at least one signaling question without information. |
| High risk: 2 or more domains have signaling questions indicating high-risk |

# **Appendix Table 5.** Risk of bias assessments for randomized studies of interventions.

| **Study** | **Bias arising from the randomization process** | | | **Risk-of-bias judgment** | **Bias due to deviations from intended interventions** | | | | | | | **Risk-of-bias judgment** | **Bias due to missing outcome data** | | | | **Risk-of-bias judgment** | **Bias in the measurement of the outcome** | | | | | **Risk-of-bias judgment** | **Bias in the selection of the reported result** | | | **Risk-of-bias judgment** | **Overall bias** |
| --- | --- | --- | --- | --- | --- | --- | --- | --- | --- | --- | --- | --- | --- | --- | --- | --- | --- | --- | --- | --- | --- | --- | --- | --- | --- | --- | --- | --- |
|  | **1.1** | **1.2** | **1.3** |  | **2.1** | **2.2** | **2.3** | **2.4** | **2.5** | **2.6** | **2.7** |  | **3.1** | **3.2** | **3.3** | **3.4** |  | **4.1** | **4.2** | **4.3** | **4.4** | **4.5** |  | **5.1** | **5.2** | **5.3** |  |  |
| Abiodun O, 2021 | Y | Y | N | Low | Y | N | PN | NI | NI | Y | NI | Low | Y | NI | NI | NI | Low | Y | N | NI | NI | NI | High | Y | N | N | Low | Some |
| Amstutz A, 2020 | NI | NI | NI | High | NI | NI | NI | NI | NI | NI | NI | High | NI | NI | NI | NI | High | NI | NI | NI | NI | NI | High | NI | NI | NI | High | High |
| Aninanya GA, 2015 | Y | Y | N | Low | Y | Y | PN | NI | NI | Y | NI | Low | N | N | N | NI | Low | PN | N | Y | N | NI | Low | Y | N | N | Low | Low |
| Bermudez LG, 2018 | PY | N | N | Low | Y | Y | N | NA | NA | Y | NA | Low | N | N | Y | Y | High | N | N | Y | N | N | Low | Y | N | N | Low | Some |
| Birdthistle I, 2022 | PY | Y | N | Some | Y | Y | PN | NI | NI | Y | NI | Low | N | N | Y | PY | High | N | N | Y | N | NI | Low | Y | N | N | Low | Low |
| Brathwaite R, 2022 | Y | PY | N | Low | N | Y | PN | NI | NI | Y | NI | Low | Y | NI | NI | NI | Low | N | N | Y | N | NI | Low | Y | N | N | Low | Low |
| Catania JA, 2021 | Y | NI | N | Low | N | Y | N | NI | NI | Y | NI | Low | Y | NI | NI | NI | Low | N | N | Y | N | NI | Low | Y | N | N | Low | Low |
| Cho H, 2018 | Y | NI | N | Low | Y | Y | PN | NI | NI | Y | NI | Low | N | N | Y | Y | High | N | N | NI | PN | NI | Low | Y | N | N | Low | High |
| Cho H, 2019 | Y | NI | N | Low | N | Y | N | NI | NI | Y | NI | Low | Y | NI | NI | NI | Low | N | N | NI | PN | NI | Low | Y | N | N | Low | Low |
| Delany-Moretlwe S, 2018 | NI | NI | NI | High | NI | NI | NI | NI | NI | NI | NI | High | NI | NI | NI | NI | High | NI | NI | NI | NI | NI | High | NI | NI | NI | High | High |
| Denison JA, 2022 | Y | Y | PN | Low | Y | Y | Y | PY | N | NI | Y | High | Y | NI | NI | NI | Low | N | N | NI | NI | NI | Low | Y | N | N | Low | High |
| Donenberg GR, 2022 | Y | NI | N | Low | Y | Y | PN | NI | NI | Y | NI | Low | Y | NI | NI | NI | Low | PN | N | NI | NI | NI | Low | Y | N | N | Low | Low |
| Dow DE, 2020 | Y | Y | N | Low | N | N | NI | NI | NI | Y | NI | Low | Y | NI | NI | NI | Low | N | N | PY | PN | NI | Low | Y | N | N | Low | Low |
| Dow DE, 2022 | Y | Y | N | Low | N | Y | N | NI | NI | Y | NI | Low | Y | NI | NI | NI | Low | N | PN | NI | PN | NI | Low | Y | N | N | Low | Low |
| Ekwunife OI, 2022 | Y | Y | N | Low | PN | Y | N | NI | NI | Y | NI | Low | Y | NI | NI | NI | Low | N | N | NI | PN | NI | Low | Y | N | N | Low | Low |
| Gitahi N, 2023 | NI | NI | NI | High | NI | NI | NI | NI | NI | NI | NI | High | NI | NI | NI | NI | High | NI | NI | NI | NI | NI | High | NI | NI | NI | High | High |
| Hallfors DD, 2015 | Y | NI | N | Low | Y | Y | Y | Y | N | Y | NI | High | N | N | NI | N | Some | Y | N | Y | N | NI | High | Y | N | N | Low | High |
| Hémono R, 2024 | Y | Y | N | Low | Y | Y | NI | NI | NI | Y | NA | Some | Y | NA | NA | NA | Low | N | N | Y | PY | NI | High | Y | N | N | Low | High |
| Hensen B, 2023 | Y | NI | N | Low | Y | Y | Y | Y | N | Y | NI | High | N | Y | NI | NI | Low | Y | N | Y | N | N | High | Y | N | N | Low | High |
| Hewett PC, 2016 | NI | NI | NI | High | NI | NI | NI | NI | NI | NI | NI | High | NI | NI | NI | NI | High | NI | NI | NI | NI | NI | High | NI | NI | NI | High | High |
| Hunter LA, 2020 | NI | NI | NI | High | NI | NI | NI | NI | NI | NI | NI | High | NI | NI | NI | NI | High | NI | NI | NI | NI | NI | High | NI | NI | NI | High | High |
| Indravudh PP, 2021 | NI | NI | NI | High | NI | NI | NI | NI | NI | NI | NI | High | NI | NI | NI | NI | High | NI | NI | NI | NI | NI | High | NI | NI | NI | High | High |
| Ketchaji A, 2019 | NI | NI | NI | High | NI | NI | NI | NI | NI | NI | NI | High | NI | NI | NI | NI | High | NI | NI | NI | NI | NI | High | NI | NI | NI | High | High |
| Kizito S, 2022 | Y | NI | N | Low | N | Y | N | NI | NI | Y | NI | Low | Y | NI | NI | NI | Low | N | N | Y | N | N | Low | Y | N | N | Low | Low |
| Kizito S, 2023 | NI | NI | NI | High | NI | NI | NI | NI | NI | NI | NI | High | NI | NI | NI | NI | High | NI | NI | NI | NI | NI | High | NI | NI | NI | High | High |
| Kizito S, 2023 | NI | NI | NI | High | NI | NI | NI | NI | NI | NI | NI | High | NI | NI | NI | NI | High | NI | NI | NI | NI | NI | High | NI | NI | NI | High | High |
| Kopo M, 2023 | Y | Y | PY | Some | PY | N | NI | NI | NI | Y | NA | Some | PY | NA | NA | NA | Low | N | N | N | NA | NA | Low | Y | N | N | Low | Some |
| Kranzer K, 2018 | PY | N | N | Low | Y | Y | N | NA | NA | Y | NA | Low | N | N | Y | Y | High | N | N | Y | N | N | Low | Y | N | N | Low | Some |
| Kuo C, 2020 | Y | NI | N | Low | Y | Y | N | NI | NI | Y | NI | Low | Y | Y | NI | NI | Low | Y | N | Y | N | N | High | Y | N | N | Low | High |
| Linnemayr S, 2017 | Y | N | N | Low | Y | Y | N | NI | NI | Y | NI | Low | Y | NI | NI | NI | Low | N | N | N | NI | NI | Low | Y | N | N | Low | Low |
| MacCarthy S, 2020 | Y | NI | N | Low | N | Y | N | NI | NI | Y | NI | Low | Y | NI | NI | NI | Low | N | N | Y | N | N | Low | Y | N | N | Low | Low |
| Mavhu W, 2020 | Y | NI | N | Low | N | Y | N | NI | NI | Y | NI | Low | Y | NI | NI | NI | Low | N | N | NI | PN | NI | Low | Y | N | N | Low | Low |
| Merrill KG, 2023 | NI | NI | N | Some | NI | Y | N | NI | NI | Y | NI | Some | NI | N | PY | NI | High | N | Y | NI | NI | NI | High | Y | N | N | Low | High |
| Musanje K, 2024 | Y | Y | N | Low | Y | Y | N | NI | NI | Y | NA | Low | N | NI | NI | NI | Some | N | N | Y | Y | NI | High | Y | N | N | Low | High |
| Nabunya P, 2024 | Y | NI | PN | Low | Y | Y | NI | NI | NI | Y | NA | Some | Y | NA | NA | NA | Low | N | N | Y | PY | NI | High | Y | N | N | Low | High |
| Nair G, 2023 | Y | Y | PN | Low | N | N | NA | NA | NA | Y | NA | Low | Y | NA | NA | NA | Low | N | N | N | NA | NA | Low | Y | N | N | Low | Low |
| Olashore AA, 2023 | Y | Y | N | Low | N | N | NA | NA | NA | Y | NA | Low | Y | NA | NA | NA | Low | N | N | Y | PY | NI | High | Y | N | N | Low | High |
| Osita EE, 2022 | Y | Y | N | Low | Y | Y | N | NI | NI | Y | NI | Low | Y | NI | NI | NI | Low | Y | N | NI | NI | NI | Some | Y | N | N | Low | Some |
| Patel RC, 2022 | Y | Y | N | Low | Y | N | NI | NI | PN | Y | NA | Some | Y | NA | NA | NA | Low | N | N | N | N | N | Low | Y | N | N | Low | Some |
| Pettifor A, 2016 | Y | Y | N | Low | Y | N | N | NI | NI | Y | NI | Low | Y | NI | NI | NI | Low | N | N | NI | PN | N | Low | Y | N | N | low | low |
| Phiri MM, 2024 | NI | Y | N | Low | Y | Y | NI | NI | NI | Y | NA | Some | PY | NA | NA | NA | Low | N | N | N | NA | NA | Low | Y | N | N | Low | Some |
| Pike C, 2023 | Y | Y | N | Low | NI | Y | Y | Y | Y | Y | NA | High | Y | NA | NA | NA | Low | N | N | Y | Y | N | Some | Y | N | N | Low | High |
| Sakthivel R, 2023 | Y | NI | N | Some | NI | NI | N | NI | NI | Y | NI | Some | Y | NI | NI | NI | Low | N | N | NI | PN | N | Low | Y | N | N | Low | Some |
| Shanaube K, 2017 | NI | NI | NI | High | NI | NI | NI | NI | NI | NI | NI | High | NI | NI | NI | NI | High | NI | NI | NI | NI | NI | High | NI | NI | NI | High | High |
| Shanaube K, 2021 | Y | NI | NI | Low | Y | Y | N | NI | NI | Y | NI | Low | N | Y | NI | NI | Low | N | N | Y | N | N | Low | Y | N | N | Low | Low |
| Simms V, 2022 | Y | N | N | Low | N | Y | N | NI | NI | Y | NI | Low | Y | NI | NI | NI | Low | N | N | Y | N | NI | Low | Y | N | N | Low | Low |
| Speizer IS, 2020 | Y | NI | N | Low | NI | NI | PN | NI | NI | Y | NI | Low | Y | NI | NI | NI | Low | N | N | NI | PN | NI | Low | PY | N | N | Low | Low |
| Speizer IS, 2020 | Y | NI | N | Low | PN | PY | PN | NI | NI | Y | NI | Low | Y | NI | NI | NI | Low | Y | N | NI | NI | NI | Some | Y | N | N | Low | Low |
| Ssewamala FM, 2020 | Y | Y | N | Low | PN | Y | N | NI | NI | Y | NI | Low | Y | NI | NI | NI | Low | N | N | PY | N | NI | Low | Y | N | N | Low | Low |
| Tozan Y, 2021 | Y | NI | N | Some | NI | NI | PN | NI | NI | Y | NI | Some | Y | NI | NI | NI | Low | N | N | NI | N | NI | Low | Y | N | N | Low | Some |
| Vreeman RC, 2019 | Y | NI | N | Some | NI | PY | N | NI | NI | Y | NI | Some | Y | NI | NI | NI | Low | Y | N | NI | NI | NI | Some | Y | N | N | Low | Some |
| Waidler J, 2022 | Y | Y | N | Low | Y | Y | N | NI | NI | Y | NI | Low | Y | NI | NI | NI | Low | Y | N | NI | NI | NI | Some | Y | N | N | Low | Some |
| Wango GN, 2023 | Y | Y | N | Low | Y | Y | N | N | NI | Y | NI | Low | Y | NI | NI | NI | Low | N | N | PN | NI | NI | Low | Y | N | N | Low | Low |
| Willis N, 2019 | Y | PY | N | Low | Y | Y | N | NI | NI | Y | NI | Low | Y | NI | NI | NI | Low | Y | Y | NI | NI | NI | High | Y | N | N | Low | Some |
| Wirsiy FS, 2022 | Y | Y | N | Low | Y | Y | N | NI | NI | Y | NI | Low | Y | NI | NI | NI | Low | N | N | N | NI | NI | Low | Y | N | N | Low | Low |
| Zanoni BC, 2024 | Y | Y | N | Low | PY | N | Y | PY | N | Y | NA | High | Y | NA | NA | NA | Low | N | N | N | NA | NA | Low | Y | N | N | Low | High |
| Zulaika G, 2023 | Y | Y | N | Low | N | N | NA | NA | NA | Y | NI | Low | Y | NA | NA | NA | Low | N | N | N | NA | NA | Low | Y | N | N | Low | Low |

# **Appendix Table 6.** Risk of bias assessments for non-randomized studies of interventions.

| **Study** | **1. Selection bias** | | | **Bias due to missing outcome data** | **Bias in the measurement of the outcome** | | | | | **Bias in analysis** | **Overall judgment of risk of bias** |
| --- | --- | --- | --- | --- | --- | --- | --- | --- | --- | --- | --- |
|  | **1.1** | **1.2** | **1.3** | **2.1** | **3.1** | **3.2** | **3.3** | **3.4** | **3.5** | **4.1** |  |
| Abili M, 2023 | Y | Y | N | N | Y | Y | Y | Y | Y | Y | High |
| Amzel A, 2018 | Y | Y | N | NI | Y | Y | N | N | Y | N | Medium |
| Arije O, 2023 | Y | Y | N | N | Y | Y | Y | NI | N | Y | High |
| Barker D, 2019 | Y | Y | Y | NI | Y | N | Y | Y | Y | Y | Medium |
| Barnabee G, 2023 | Y | N | N | Y | Y | Y | Y | Y | Y | Y | High |
| Beck-Sague CM, 2015 | Y | Y | N | NI | Y | Y | Y | Y | N | N | High |
| Birdthistle I, 2022 | Y | Y | NI | NI | Y | Y | Y | Y | NI | N | High |
| Casalini C, 2023 | Y | Y | N | Y | Y | Y | Y | Y | Y | NI | Medium |
| Chimbindi N, 2023 | Y | NI | N | Y | Y | Y | NI | Y | Y | Y | Medium |
| Ciccaci F, 2023 | Y | Y | N | Y | Y | Y | Y | Y | Y | Y | Medium |
| Dhakwa D, 2021 | Y | Y | Y | NI | N | Y | N | Y | Y | Y | High |
| Dougherty G, 2022 | Y | Y | Y | N | NI | Y | N | NI | NI | Y | High |
| Floyd S, 2018 | N | NI | N | N | Y | Y | Y | Y | Y | Y | High |
| Galárraga O, 2020 | Y | Y | Y | NI | Y | Y | Y | Y | Y | Y | Medium |
| Jani N, 2016 | Y | Y | Y | Y | Y | Y | Y | Y | NI | Y | Medium |
| Jubilee M, 2019 | N | Y | Y | Y | Y | Y | Y | Y | Y | Y | Medium |
| Kabogo J, 2018 | Y | Y | N | NI | Y | Y | Y | Y | NI | N | Medium |
| Kitetele FN, 2023 | Y | N | NI | Y | Y | N | Y | Y | Y | Y | High |
| Kose J, 2018 | Y | Y | N | N | N | Y | Y | N | Y | N | High |
| Kubheka SE, 2020 | N | Y | N | Y | Y | Y | Y | Y | Y | Y | High |
| Letsela L, 2021 | Y | Y | N | NI | NI | NI | NI | NI | NI | NI | Low |
| Levy M, 2021 | Y | Y | Y | Y | NI | Y | Y | Y | Y | NI | Low |
| Mackenzie RK, 2017 | Y | Y | N | NI | N | Y | Y | Y | Y | NI | Low |
| Massa P, 2023 | Y | Y | Y | Y | Y | Y | Y | Y | Y | Y | Low |
| Mathur S, 2022 | Y | NI | N | Y | Y | Y | NI | Y | Y | Y | Medium |
| Menna T, 2015 | Y | Y | Y | N | Y | Y | Y | Y | Y | Y | Medium |
| Mthiyane N, 2022 | Y | Y | N | Y | Y | Y | Y | Y | Y | Y | Medium |
| Muchabaiwa L, 2018 | Y | Y | Y | N | Y | Y | Y | Y | N | Y | High |
| Mulwa S, 2021 | N | Y | Y | Y | N | Y | Y | Y | Y | NI | Medium |
| Munyayi FK, 2020 | Y | Y | Y | N | Y | Y | Y | Y | Y | Y | Medium |
| Munyayi FK, 2020 | Y | Y | Y | Y | Y | Y | Y | Y | Y | Y | Low |
| Ness TE, 2021 | Y | Y | Y | Y | Y | Y | Y | Y | N | Y | Medium |
| Nwanja E, 2023 | Y | Y | Y | Y | Y | Y | Y | N | Y | Y | Medium |
| Oberth G, 2021 | N | N | N | Y | Y | Y | Y | Y | Y | Y | High |
| Rucinski K, 2022 | Y | Y | Y | NI | Y | Y | Y | Y | Y | Y | Medium |
| Ruria EC, 2017 | Y | NI | Y | Y | Y | Y | Y | Y | Y | Y | Low |
| Stangl AL, 2021 | Y | NI | N | Y | Y | Y | Y | Y | Y | NI | Medium |
| Thurman TR, 2024 | Y | Y | Y | Y | Y | Y | Y | Y | Y | Y | Low |
| Trapence CP, 2023 | Y | NI | Y | N | Y | Y | NI | Y | Y | Y | Medium |
| Tunje A, 2024 | Y | Y | N | Y | Y | Y | Y | Y | Y | Y | Medium |
| Tymejczyk O, 2020 | Y | Y | N | NI | N | Y | Y | Y | Y | Y | High |
| Yumo HA, 2018 | Y | Y | Y | N | N | Y | Y | Y | Y | Y | High |

# **Appendix Table 7.** Effect of different levels of adolescent engagement on the outcomes across the HIV prevention and care cascade and related social determinants outcomes.

|  | **PrEP uptake** | | | **HIV testing** | | | **Awareness of HIV infection** | | | **ART adherence** | | | **ART retention** | | | **Virological suppression** | | |
| --- | --- | --- | --- | --- | --- | --- | --- | --- | --- | --- | --- | --- | --- | --- | --- | --- | --- | --- |
|  | Number of studies (RCTs; non-randomized trials) | Risk ratio (95% CI) | I² | Number of studies (RCTs; non-randomized trials) | Risk ratio (95% CI) | I² | Number of studies (RCTs; non-randomized trials) | Risk ratio (95% CI) | I² | Number of studies (RCTs; non-randomized trials) | Risk ratio (95% CI) | I² | Number of studies (RCTs; non-randomized trials) | Risk ratio (95% CI) | I² | Number of studies (RCTs; non-randomized trials) | Risk ratio (95% CI) | I² |
| No/Minimum engagement | .. | .. | .. | 13 (8, 5) | 1.23 (1.15-1.31) | 98.5% | 4 (3, 1) | 2.77 (2.74-2.80) | 99.5% | 12 (6, 6) | 1.15 (1.02-1.28) | 76.9% | 4 (2, 2) | 1.23 (1.15-1.30) | 90.5% | 14 (5, 9) | 1.20 (1.17-1.23) | 96.1% |
| Moderate/substantial engagement | 3 (2, 1) | 1.82 (1.24-2.66) | 41.5% | 9 (5, 4) | 2.37 (1.43-3.93) | 99.8% | 3 (1, 2) | 1.82 (1.74-1.90) | 98.1% | 2 (0, 2) | 1.13 (0.91-1.39) | 52.2% | 4 (1, 3) | 1.06 (1.03-1.09) | 41.8% | 12 (7, 5) | 1.04 (1.01-1.07) | 27.4% |

|  | **Violence experience** | | | **School drop-out** | | | | **Total stigma** | | | | **Self-stigma** | | | | **External stigma** | | |
| --- | --- | --- | --- | --- | --- | --- | --- | --- | --- | --- | --- | --- | --- | --- | --- | --- | --- | --- |
|  | Number of studies (RCTs; non-randomized trials) | Risk ratio (95% CI) | I² | Number of studies (RCTs; non-randomized trials) | Risk ratio (95% CI) | I² | Number of studies (RCTs; non-randomized trials) | | SMD (95% CI) | I² | Number of studies (RCTs; non-randomized trials) | | SMD (95% CI) | I² | Number of studies (RCTs; non-randomized trials) | | SMD (95% CI) | I² |
|  |
| Minimum engagement | 2 (1, 1) | 0.82 (0.67-1.02) | 0.0% | 4 (3, 1) | 0.46 (0.24-0.86) | 85.9% | 1 (0, 1) | | -0.27(-0.75-0.22) | .. | .. | | .. | .. | .. | | .. | .. |  |
| Moderate/substantial engagement | 2 (1, 1) | 0.71 (0.55-0.91) | 92.3% | 2 (1, 1) | 0.55 (0.20-1.51) | 97.0% | 3 (2, 1) | | 0.11 (-0.14-0.37) | 0.0% | 4 (2, 2) | | -0·25 (-0.53, 0.03) | 30.60% | 3 (2, 1) | | -0.05 (-0.46, 0.36) | 64.7% |  |

|  | **Transactional sex** | | | **Condom usage** | | | **Pregnancy** | | | **STI co-infection** | | | **Utilize sexual reproductive health service** | | |
| --- | --- | --- | --- | --- | --- | --- | --- | --- | --- | --- | --- | --- | --- | --- | --- |
|  | Number of studies (RCTs; non-randomized trials) | Risk ratio (95% CI) | I² | Number of studies (RCTs; non-randomized trials) | Risk ratio (95% CI) | I² | Number of studies (RCTs; non-randomized trials) | Risk ratio (95% CI) | I² | Number of studies (RCTs; non-randomized trials) | Risk ratio (95% CI) | I² | Number of studies (RCTs; non-randomized trials) | Risk ratio (95% CI) | I² |
| Minimum engagement | 1 (1, 0) | 0.53 (0.32-0.89) | .. | 2 (1, 1) | 1.11 (0.98-1.26) | 45.8% | 3 (3, 0) | 0.61 (0.39-0.93) | 0.0% | 3 (3, 0) | 1.37 (1.01-1.86) | 0.0% | 3 (1, 2) | 1.14 (0.96-1.34) | 74.7% |
| Moderate/substantial engagement | 2 (1, 1) | 1.24 (0.80-1.93) | 64.4% | 7 (4, 3) | 1.01 (0.94-1.08) | 79.3% | 4 (3, 1) | 1.06 (0.83-1.36) | 85.8% | 5 (3, 2) | 0.93 (0.82-1.06) | 31.2% | 4 (3, 1) | 2.29 (1.52-3.45) | 96.0% |

# **Appendix Figure 1.** Forest plot examining the association between interventions and PrEP uptake.


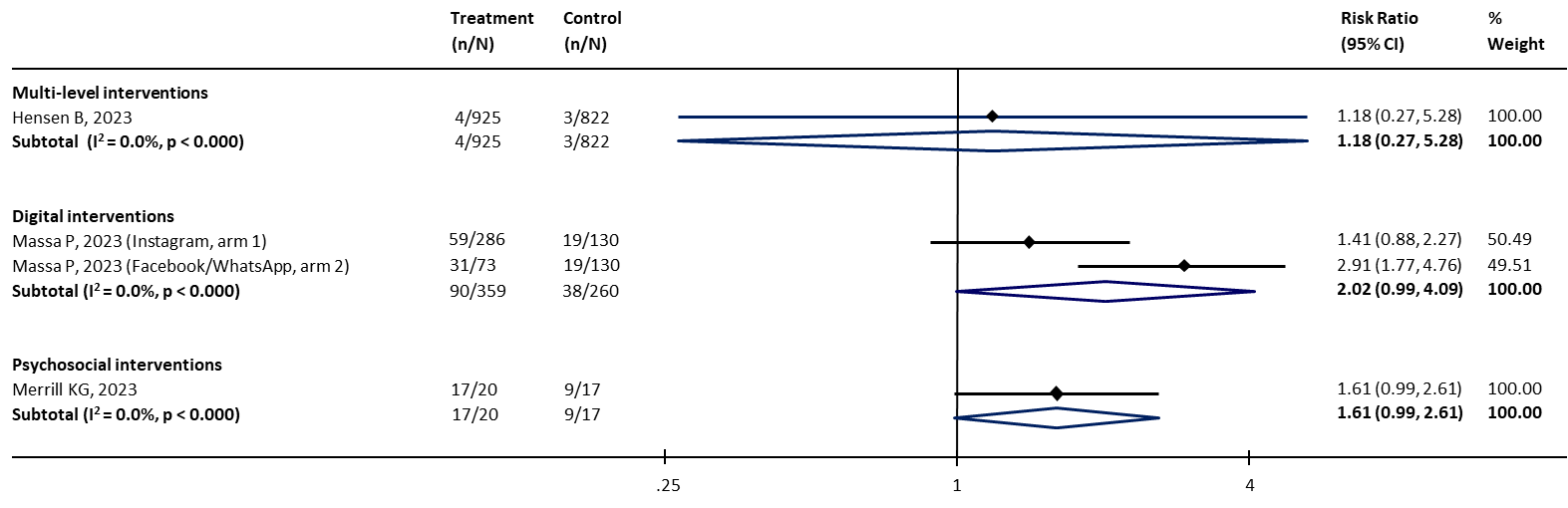


# **Appendix Figure 2.** Funnel plot assessing publication bias for intervention studies on PrEP uptake. The Egger’s test indicates that no publication bias is present (p=0.784).

# **Appendix Figure 3.** Funnel plot assessing publication bias for intervention studies on HIV testing. The Egger’s test indicates that publication bias is present (p=0.002).

# **Appendix Figure 4.** Funnel plot assessing publication bias for intervention studies on people’s awareness of HIV infections. The Egger’s test indicates that no publication bias is present (p=0.490).

# **Appendix Figure 5.** Funnel plot assessing publication bias for intervention studies on ART adherence. The Egger’s test indicates that no publication bias is present (p=0.173).

# **Appendix Figure 6.** Funnel plot assessing publication bias for intervention studies on retention in care. The Egger’s test indicates that no publication bias is present (p=0.137).

# **Appendix Figure 7.** Funnel plot assessing publication bias for intervention studies on virological suppression. The Egger’s test indicates that no publication bias is present (p=0.166).

**
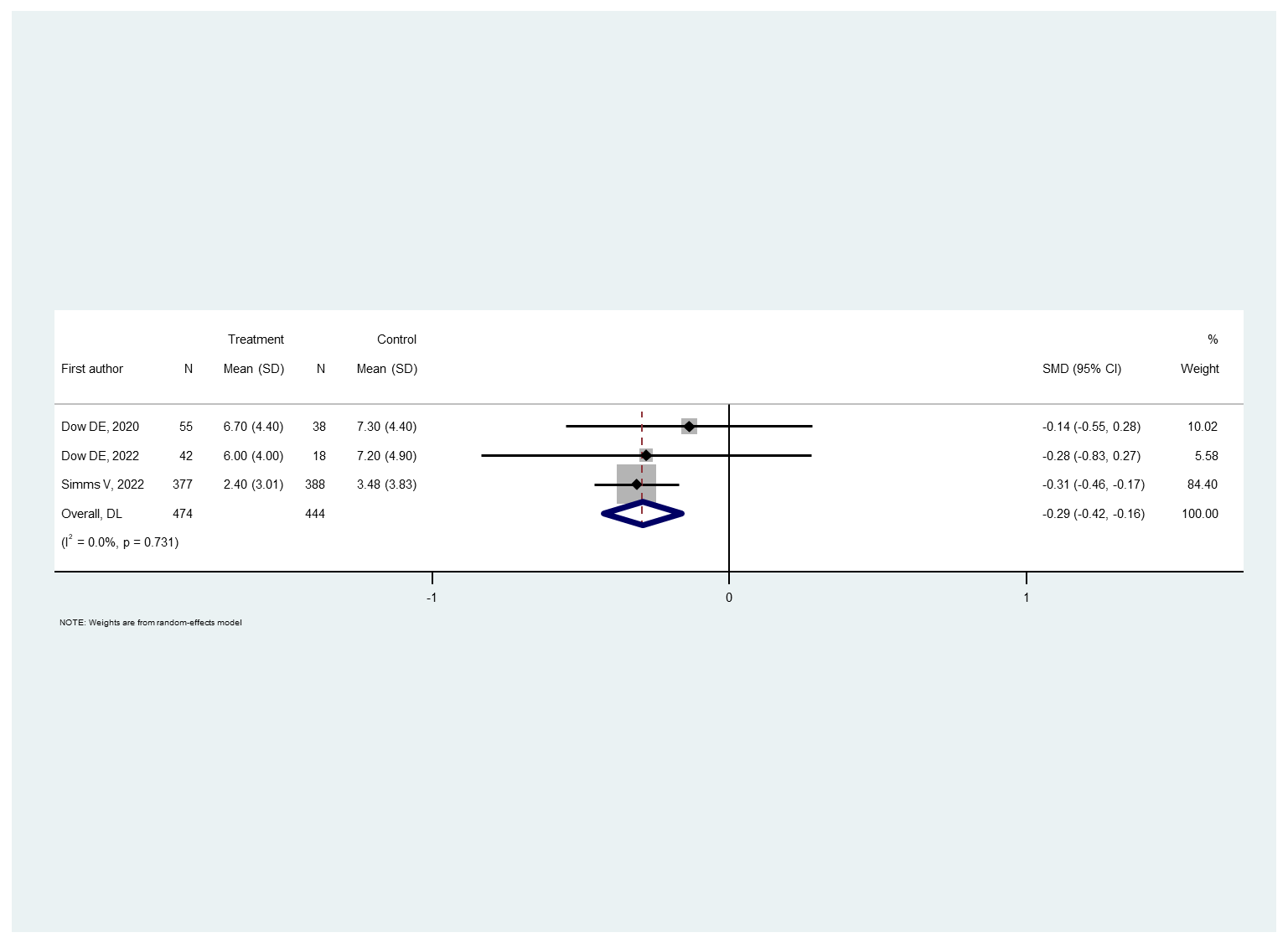
Appendix Figure 8.** Forest plot examining the association between interventions and symptoms of mental health problems.

# **Appendix Figure 9.** Forest plot examining the association between interventions and violence experience.

.

.

.

Mathur S, 2022 (Kenya)

Mathur S, 2022 (Malawian)

Mulwa S, 2021

ID

Economic Strengthening

Mathur S, 2022 (Zambia)

Pettifor A, 2016

Subtotal (I-squared = 89.1%, p = 0.000)

Study

Self-care

Wango GN, 2023

Subtotal (I-squared = .%, p = .)

Asset building

Subtotal (I-squared = .%, p = .)

0.72 (0.62, 0.84)

0.52 (0.42, 0.66)

0.83 (0.67, 1.02)

RR (95% CI)

0.99 (0.88, 1.11)

0.64 (0.58, 0.71)

0.75 (0.58, 0.97)

0.66 (0.11, 3.89)

0.66 (0.11, 3.89)

0.64 (0.58, 0.71)

.

26.06

23.20

23.81

Weight

26.94

100.00

100.00

%

100.00

100.00

100.00

0.72 (0.62, 0.84)

0.52 (0.42, 0.66)

0.83 (0.67, 1.02)

RR (95% CI)

0.99 (0.88, 1.11)

0.64 (0.58, 0.71)

0.75 (0.58, 0.97)

0.66 (0.11, 3.89)

0.66 (0.11, 3.89)

0.64 (0.58, 0.71)

.

26.06

23.20

23.81

Weight

26.94

100.00

100.00

%

100.00

100.00

100.00

1

.112

1

8.97

# **Appendix Figure 10.** Forest plot examining the association between interventions and school drop-out.

# **
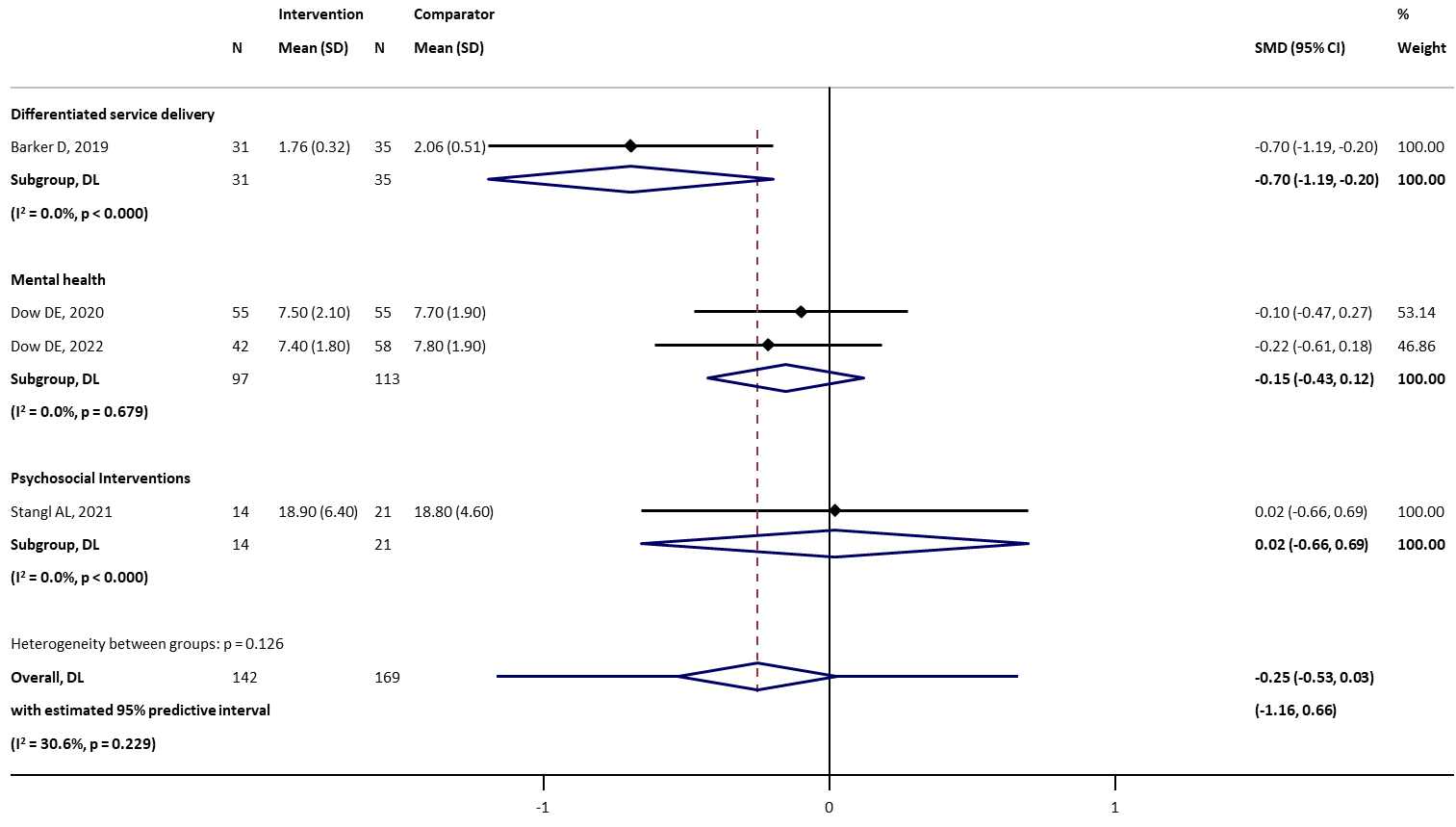
Appendix Figure 11.** Forest plot examining the association between interventions and self-stigma.

# **
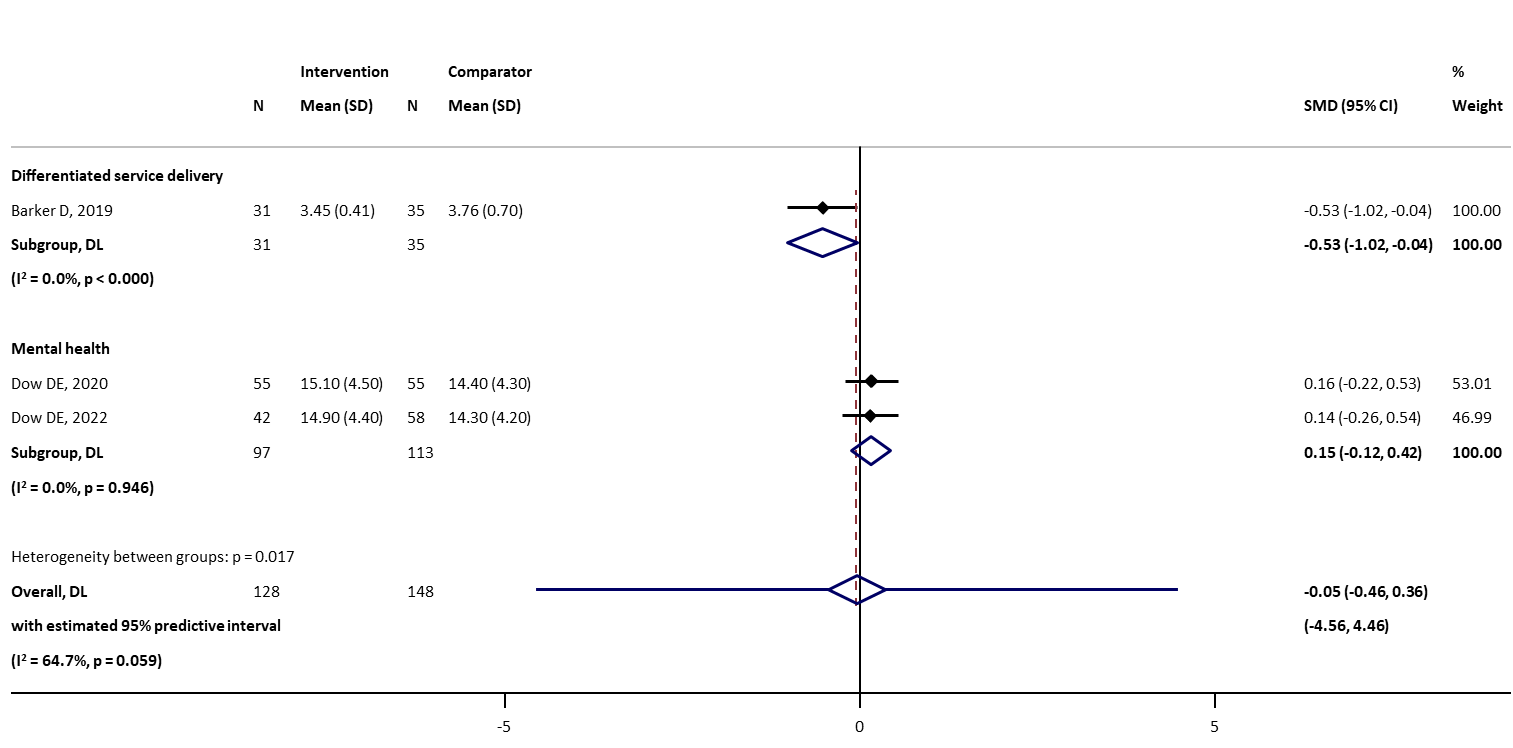
Appendix Figure 12.** Forest plot examining the association between interventions and experienced stigma.

# **Appendix Figure 13.** Forest plot examining the association between interventions and transactional sex.

# **Appendix Figure 14.** Forest plot examining the association between interventions and condom usage.

# **Appendix Figure 15.** Forest plot examining the association between interventions and adolescent pregnancy.

# **Appendix Figure 16.** Forest plot examining the association between interventions and STI Co-infection.

# **Appendix Figure 17.** Forest plot examining the association between interventions and sexual and reproductive health service utilization.
